# Sarcopenia and Its Associated Metabolic Profile Predict Incident Heart Failure: A Prospective Cohort Study of 267 335 Adults in the UK Biobank

**DOI:** 10.1161/JAHA.125.047621

**Published:** 2026-07-17

**Authors:** Ziyi Zhong, Yang Chen, Qiao Xiang, Hongyu Liu, Manlin Zhao, Vanja Pekovic‐Vaughan, Reijo Sund, Rajiv Sankaranarayanan, Daniel J. Cuthbertson, Masoud Isanejad

**Affiliations:** ^1^ Department of Musculoskeletal and Ageing Science, Institute of Life Course and Medical Sciences University of Liverpool Liverpool UK; ^2^ Department of Cardiovascular and Metabolic Medicine, Institute of Life Course and Medical Sciences University of Liverpool Liverpool UK; ^3^ Liverpool Centre for Cardiovascular Science at University of Liverpool Liverpool John Moores University and Liverpool Heart & Chest Hospital Liverpool UK; ^4^ Institute of Clinical Medicine, Kuopio Musculoskeletal Research Unit University of Eastern Finland Kuopio Finland; ^5^ Liverpool University Hospitals NHS Foundation Trust Aintree Hospital Liverpool Merseyside UK; ^6^ Metabolism & Nutrition Research Group Liverpool University Hospitals NHS Foundation Trust Liverpool UK

**Keywords:** heart failure, model discrimination, NMR metabolomics, sarcopenia, UK Biobank, Heart Failure, Aging, Cardiovascular Disease, Epidemiology, Women

## Abstract

**Background:**

Sarcopenia is prevalent in heart failure (HF), but its role in incident HF and underlying metabolic mechanisms remains unclear. We examined the interplay between sarcopenia phenotypes, circulating metabolic profiles, and incident HF.

**Methods:**

We analyzed UK Biobank participants without baseline HF. Associations between sarcopenia phenotypes and HF incidence were assessed using Cox regression. Nuclear magnetic resonance metabolomics was used to characterize sarcopenia‐related profiles. Cox regression was applied to test metabolite–HF associations, and least absolute shrinkage and selection operator regression was further used to refine predictors. Incremental predictive value beyond clinical risk factors was evaluated using discrimination and reclassification metrics.

**Results:**

During a median 15.3 years, 10 233 of 267 335 participants (mean age 56.5 ± 8.1 years; 44.6% men) developed HF. Groups with confirmed sarcopenia (n=1993) and low handgrip strength (normalized to body mass index) only (n=18 796) were associated with higher HF risk (hazard ratio [HR], 1.63 [95% CI, 1.44–1.85]; HR, 1.76 [95% CI, 1.66–1.85]) compared with the reference group, with stronger effects observed in younger adults and women. Metabolomic profiling revealed sarcopenia‐related alterations (higher glycoprotein acetyls, glucose–lactate, phenylalanine, tyrosine, 3‐hydroxybutyrate; lower omega‐3 fatty acids, docosahexaenoic acid, glycine, glutamine, histidine), which also predicted higher HF risk (Bonferroni‐adjusted *P* < 0.05). Selected metabolites are significant mediators and modestly improved HF prediction (15‐year net reclassification improvement 14%–15%; integrated discrimination improvement 0.9%–1.0%).

**Conclusions:**

Sarcopenia, particularly reduced handgrip strength, was a strong predictor for incident HF. Lipid‐, amino acid‐, and energy metabolism–related alterations modestly improved risk prediction and partially mediated the association.

**Nonstandard Abbreviations and Acronyms**
ALM: appendicular lean mass
FNIH: Foundation for the National Institutes of Health
HGS: handgrip strength
IDI: integrated discrimination improvement
LASSO: least absolute shrinkage and selection operator
NMR: nuclear magnetic resonance
NRI: net reclassification improvement
UKB: UK Biobank

**Clinical Perspective**

**What Is New?**

- In this large prospective cohort of 267 335 adults, Foundation for the National Institutes of Health‐defined sarcopenia, particularly reduced handgrip strength, was independently associated with a substantially higher risk of incident heart failure over 15 years of follow‐up.
- Sarcopenia‐related metabolic signatures characterized by inflammatory, lipid, amino acid, and energy metabolism alterations were prospectively associated with higher heart failure risk and partially mediated the associations.

**What Are the Clinical Implications?**

- Simple assessment of muscle strength and targeted metabolomic profiling may improve heart failure risk stratification and support earlier preventive strategies focused on skeletal muscle health.

Heart failure (HF) is a complex clinical syndrome characterized by heterogeneous pathophysiology, and despite advances in treatment, prognosis remains poor.^1^, ^2^ Approximately 56 million people globally and 1 million people in the United Kingdom are diagnosed with HF.^3^ Excess adiposity and obesity are well‐known risk factors for HF,^4^ through a myriad of mechanisms.^5^ Excess adiposity promotes intramuscular and myocardial fat deposition, which may exacerbate sarcopenia (a condition characterized by reduced skeletal muscle mass and strength), impair cardiac function, and increase HF risk.^6^, ^7^ Sarcopenia is increasingly recognized as a key noncardiac contributor to adverse prognosis of HF,^8^ being associated with reduced physical function and exercise intolerance and affecting approximately half of patients with HF.^9^ However, despite its high prevalence in HF, it remains unclear whether sarcopenia can precede HF development or is merely an associated clinical feature of HF, leaving the temporal sequence uncertain. Although previous studies have examined 1 component of sarcopenia, reduced handgrip strength (HGS), in relation to incident HF,^10^, ^11^ they were limited by short follow‐up durations and lack of mechanistic exploration. Therefore, we also attempted to strengthen the biological novelty of the temporal associations through metabolomics approach to provide further mechanistic insight compared with previous work.

Metabolic dysregulation has been established as a hallmark of HF and is associated with an adverse prognosis and treatment outcomes.^12^ Beyond myocardial energetic failure, metabolic dysregulation increasingly points to peripheral and systemic alterations as key mechanisms in the development of HF.^13^ However, most studies have focused predominantly on cardiac metabolism, with limited investigation of the active role of other metabolically active organs, particularly skeletal muscle. Given the high prevalence of sarcopenia and reduced exercise capacity in HF, it is plausible that some of these dysregulated skeletal muscle metabolic pathways may overlap with, or even precede, the development of HF. For example, circulating branched‐chain amino acids, histidine, ketone bodies, phenylalanine, tyrosine, and glycolytic metabolites are increasingly linked to HF progression,^14^, ^15^ and a number of these have also been associated with muscle weakness.^16^, ^17^ These overlapping and characteristic metabolic signatures raise the possibility that systemic metabolic remodeling may partly underlie the association between sarcopenia and HF. Given that direct mechanistic studies have shown impaired skeletal muscle metabolism in HF,^18^ we hypothesize that sarcopenia‐related metabolic alterations may contribute to an increased risk of HF. Advances in metabolomics now enable large‐scale profiling of circulating metabolites, providing molecular insights into disease mechanisms.

In this study, we leveraged a large, prospective UKB (UK Biobank) cohort to address these knowledge gaps. First, we investigated the associations between sarcopenia and incident HF. Second, we delineated their characteristic metabolomic signatures and examined the prospective associations of sarcopenia‐related metabolites with HF risk. Finally, we evaluated whether selected metabolites enhanced risk prediction beyond established clinical cardiovascular risk factors and explored their potential mediating role. These results could inform strategies for HF prevention related to impaired skeletal muscle health.

## METHODS

### Data Availability

Data are available from the UKB (http://www.ukbiobank.ac.uk) subject to approval. Additional materials supporting this study are available from the corresponding authors upon reasonable request.

### Study Design and Population

This study was conducted within the UKB, a population‐based prospective cohort of >500 000 participants aged 37 to 73 years recruited between 2006 and 2010 across England, Scotland, and Wales. Detailed study protocols have been published previously and are available online (https://www.ukbiobank.ac.uk). Ethical approval was granted by the Northwest Multi‐centre Research Ethics Committee (REC reference: 11/NW/0382), and all participants provided written informed consent.

We included participants with baseline measurements of HGS, fat‐free mass by bioelectrical impedance analysis, body mass index (BMI), and plasma nuclear magnetic resonance (NMR)‐based metabolomics. Those with a prior HF diagnosis before baseline was excluded, leaving 267 335 participants for analysis (Figure S1).

We conducted a 2‐part analysis. The first part assessed associations between sarcopenia phenotypes (definitions provided subsequently) and incident HF. The second metabolomics part comprised 2 phases. In phase 1, metabolites associated with each sarcopenia phenotype compared with the reference group were identified. In phase 2, these metabolites were tested for prospective associations with HF and incremental predictive value beyond traditional clinical risk factors.

### Sarcopenia Phenotypes Ascertainment

Measurement of HGS and appendicular lean mass (ALM) was based on cutoff points from 2 widely used international guidelines: the Foundation for the National Institutes of Health (FNIH),^19^ which considers body size–adjusted (relative) indicators and served as the primary definition given its relevance to cardiometabolic risk, and the European Working Group on Sarcopenia in Older People 2,^7^ which uses absolute HGS and only height–adjusted ALM thresholds and was applied for complementary analyses. Details are described in Data S1. Participants were categorized into 4 mutually exclusive phenotypes per definition: (1) reference (both ALM and HGS normal), (2) low ALM only (low ALM with normal HGS), (3) low HGS only (low HGS with normal ALM), and (4) confirmed sarcopenia (both low ALM and low HGS).

Sarcopenic obesity was defined as the presence of sarcopenia according to either criterion in combination with general obesity (BMI ≥30 kg/m^2^) or abdominal obesity (waist circumference ≥94 cm for men, ≥80 cm for women).^20^

### Heart Failure Diagnosis

Incident HF was identified using the UKB “First Occurrence” data with the *International Classification of Diseases, Tenth Revision* (*ICD‐10*) code of I50 (Field ID 131355), incorporating primary care, hospital episode statistics, death registries and baseline self‐reported medical condition. The event was defined as the earliest date recorded among these sources. Follow‐up time was from baseline to first HF diagnosis, death, loss to follow‐up, or administrative censoring (at the last date of linkage across all sources), whichever came first. HF subtypes were approximated using magnetic resonance imaging‐derived ventricular ejection fraction (EF) (Imaging visit), with <40% as reduced and ≥ 50% as preserved.

### Plasma Biomarker Profiling by NMR

Baseline plasma metabolites were profiled using a high‐throughput proton NMR spectroscopy platform (Nightingale Health Ltd, Helsinki, Finland). Of the 249 metabolic features quantified, we restricted analyses to 168 biomarkers available in absolute concentrations. Only baseline measures were included. Metabolites with >10% missingness would have been excluded; all selected 168 biomarkers met this threshold and were retained. Remaining missing values were imputed as half of the minimum observed value for each metabolite. More technical details are provided in Data S2.

### Covariates

Potential confounders were selected a priori based on established risk factors,^11^, ^21^ including (1) sociodemographic variables—age, sex, race (White/others [Asian or Asian British, Black or Black British, Chinese, Mixed, and Other ethnic group]), education, and Townsend deprivation index; (2) lifestyle factors—numbers of total weekly protein servings, physical activity (metabolic equivalent task minutes for all exercise per week), smoking status (never, previous, current), and alcohol intake frequency; and (3) health conditions at baseline—diabetes, hypertension, coronary heart disease, and chronic kidney disease as defined at baseline. Variable definitions and missingness summary are provided in Tables S1 and S2.

### Statistical Analysis

Baseline characteristics were summarized using means±SD or frequencies (percentages) and compared between groups using independent *t* ‐tests for continuous variables and chi‐square tests for categorical variables. Cox proportional hazards models estimated hazard ratios (HRs) and 95% CIs for sarcopenia phenotypes in relation to incident HF, using the both‐normal group as the reference category. Proportional hazards assumptions were examined using Schoenfeld residuals. Three models were fitted: Model 1 (unadjusted), Model 2 (adjusted for sociodemographic factors), and Model 3 (further adjusted for lifestyle and clinical factors). Missing covariates were imputed using multiple imputation by chained equations (details in Data S3). Cumulative incidence of HF was estimated with the Aalen–Johansen method, treating death as a competing event. Incidence rates were calculated as the number of events per 1000 person‐years. Multiplicative interaction terms were used to investigate whether the associations between sarcopenia phenotypes and incident HF differed according to age (<60 versus ≥60 years), sex (male versus female), BMI (normal [<24.9 kg/m^2^], overweight [25.0–29.9 kg/m^2^], or obese [≥30.0 kg/m^2^]), and waist circumference (abdominal obesity [≥94 cm for men and ≥ 80 cm for women] versus nonabdominal obesity), and corresponding subgroup analyses were performed. Because significant interactions with age and sex were observed, Cox models including interaction terms between sarcopenia phenotypes and age or sex were further fitted. Marginal predicted risks were derived from these models to visualize effect modification across age–sex strata. Nonlinear associations were assessed using restricted cubic spline models with knots at the 5th, 35th, 65th, and 95th percentiles. To assess threshold effects, sex‐specific segmented Cox proportional hazards models were fitted using the “segmented” package to estimate inflection points, with model fit compared using likelihood ratio tests between models with and without segmented terms. Associations below and above the identified inflection point were then estimated. Interaction and stratified analyses were additionally performed using dichotomized sarcopenia indicators based on the identified thresholds. Logistic regression models were additionally applied to examine associations between sarcopenia indicators and HF subtypes defined by left ventricular ejection fraction (HF with reduced EF versus HF with preserved EF).

For metabolomics, differential metabolite analysis was performed using covariate‐adjusted linear models implemented in the *limma* R package, applying false discovery rate and fold‐change thresholds. Pathway enrichment was subsequently assessed using MetaboAnalyst 6.0. Results were visualized with volcano and bubble plots. Cox regression was applied to examine metabolite associations with HF, and significant metabolites were further selected using least absolute shrinkage and selection operator (LASSO) to derive parsimonious predictive signatures. The penalty parameter (λ) was optimized via 10‐fold cross‐validation repeated 1000 times, and metabolites consistently selected in >95% of iterations were retained.^22^ Incremental predictive value beyond full set of clinical covariates (traditional risk factors, base model) was evaluated using Harrell's C‐index, 15‐year continuous net reclassification improvement (NRI) and integrated discrimination improvement (IDI), with 1000 bootstrap resamples. More details are provided in Data S4. Mediation analyses were conducted to evaluate the potential mediating effects of LASSO‐selected metabolites on the associations between sarcopenia phenotypes and incident HF (R package “*CMAverse*”), with 500 bootstrap resamples. Model calibration at 15 years was further assessed using decile‐based plots of observed versus predicted HF risk derived from 10‐fold cross‐validation. Calibration slopes and 95% CIs were estimated across folds and pooled using inverse variance weighting.

Several additional analyses were conducted to examine robustness. First, clinical associations were examined using 2 established sarcopenia definitions.^7^, ^19^ Second, complete‐case analyses were performed without multiple imputation. Third, to mitigate reverse causality, outcomes occurring within 2 years of baseline were excluded and primary analyses repeated. Fourth, repeat‐assessment NMR data were leveraged: baseline muscle phenotypes were related to follow‐up metabolite levels at Instance 1 for low HGS/BMI‐only phenotype, and cross‐sectional metabolomic comparisons were replicated at Instance 1 for confirmed sarcopenia given limited longitudinal cases (N=69), adjusting for the same covariates as in the primary analysis. Fifth, interaction and subgroup analyses were conducted across age, sex, BMI, and waist circumference categories. Finally, alternative thresholds for metabolite selection were applied to assess the stability of metabolomic findings.

All statistical analyses were conducted in R version 4.4.0. Statistical significance was defined as false discovery rate‐adjusted *P* < 0.05 (Benjamini–Hochberg) or Bonferroni‐adjusted *P* < 0.05 for metabolomics analyses, and 2‐sided *P* < 0.05 for all other tests. Unless otherwise stated, models were adjusted for the full set of covariates.

## RESULTS

### Baseline Characteristics

A total of 267 335 participants were included in the analysis cohort, of whom 10 233 (3.8%) developed incident HF during a median follow‐up of 15.3 years (interquartile range, 14.4–16.0) (Table S3). At baseline, 243858 (91.2%) were classified as both normal, 2688 (1.0%) as low ALM/BMI only, 18 796 (7.0%) as low HGS/BMI only, and 1993 (0.7%) as confirmed sarcopenia according to the FNIH definition (Table 1). Participants with adverse sarcopenia phenotypes were generally older, had higher adiposity, lower physical activity, and a higher burden of comorbidities than those with normal muscle status (all *P* < 0.001).

**Table 1:** Baseline Characteristics of Participants by Sarcopenia Status

	Both normal (N=243 858)	Low ALM/BMI only (N=2688)	Low HGS/BMI only (N=18 796)	Confirmed sarcopenia (N=1993)	*P* value
Sociodemographic characteristics					
Age, y	56.21 (8.09)	61.91 (6.30)	59.26 (7.32)	60.69 (6.92)	<0.001
Sex, male, n (%)	111 337 (45.7)	2059 (76.6)	7905 (42.1)	1201 (60.3)	<0.001
Race, White, n (%)	231 519 (94.9)	2439 (90.7)	17 219 (91.6)	1721 (86.4)	<0.001
Education, college or university degree (%)	80 314 (32.9)	440 (16.4)	3884 (20.7)	267 (13.4)	<0.001
Townsend Deprivation Index	−1.47 (3.01)	−0.38 (3.38)	−0.45 (3.37)	0.40 (3.65)	<0.001
Clinical measurements					
BMI, kg/m^2^	26.94 (4.27)	31.52 (5.29)	32.23 (6.17)	36.86 (7.06)	<0.001
BMI group					<0.001
BMI normal (%)	85 920 (35.2)	183 (6.8)	1820 (9.7)	29 (1.5)	
BMI overweight (%)	107 360 (44.0)	1018 (37.9)	5610 (29.8)	270 (13.5)	
BMI obesity (%)	50 578 (20.7)	1487 (55.3)	11 366 (60.5)	1694 (85.0)	
Waist circumference, cm	89.08 (12.67)	99.94 (10.91)	101.29 (15.49)	109.65 (14.09)	<0.001
Waist circumference group					<0.001
Nonabdominal obesity (%)	170 594 (70.0)	1325 (49.3)	5781 (30.8)	359 (18.0)	
Abdominal obese (%)	73 231 (30.0)	1361 (50.7)	13 006 (69.2)	1634 (82.0)	
Lifestyle factors					
Numbers of total weekly protein servings	3.70 (1.65)	3.77 (1.72)	3.66 (1.81)	3.60 (1.91)	<0.001
Summed metabolic equivalent of task min/wk for PA	2714.62 (2683.45)	2601.24 (2763.02)	2257.98 (2529.22)	2136.84 (2518.62)	<0.001
Smoking status (%)					<0.001
Current	25 814 (10.6)	304 (11.3)	1870 (9.9)	169 (8.5)	
Previous	83 607 (34.3)	1262 (46.9)	7054 (37.5)	855 (42.9)	
Never	134 437 (55.1)	1122 (41.7)	9872 (52.5)	969 (48.6)	
Alcohol frequency (%)					<0.001
Daily or almost daily	50 672 (20.8)	563 (20.9)	2474 (13.2)	249 (12.5)	
Three or four times a wk	58 516 (24.0)	525 (19.5)	3008 (16.0)	287 (14.4)	
Once or twice a wk	63 982 (26.2)	719 (26.7)	4709 (25.1)	500 (25.1)	
One to three times a mo	27 077 (11.1)	255 (9.5)	2296 (12.2)	235 (11.8)	
Special occasions only	26 197 (10.7)	338 (12.6)	3457 (18.4)	369 (18.5)	
Never	17 414 (7.1)	288 (10.7)	2852 (15.2)	353 (17.7)	
Comorbidities					
Diabetes (%)	10 108 (4.1)	291 (10.8)	2732 (14.5)	430 (21.6)	<0.001
Hypertension (%)	60 688 (24.9)	1284 (47.8)	8278 (44.0)	1129 (56.6)	<0.001
Coronary heart disease (%)	10 800 (4.4)	383 (14.2)	2022 (10.8)	350 (17.6)	<0.001
Chronic kidney disease (%)	4996 (2.0)	108 (4.0)	834 (4.4)	106 (5.3)	<0.001
Heart failure event (%)					<0.001
No	235 870 (96.7)	2435 (90.6)	17 057 (90.7)	1740 (87.3)	
Yes	7988 (3.3)	253 (9.4)	1739 (9.3)	253 (12.7)	

Data were shown as mean±SD or n (%).

ALM indicates appendicular lean mass; BMI, body mass index; HGS, handgrip strength; and PA, physical activity.

### Associations Between Sarcopenia Phenotypes and Incident HF

**Figure 1:**
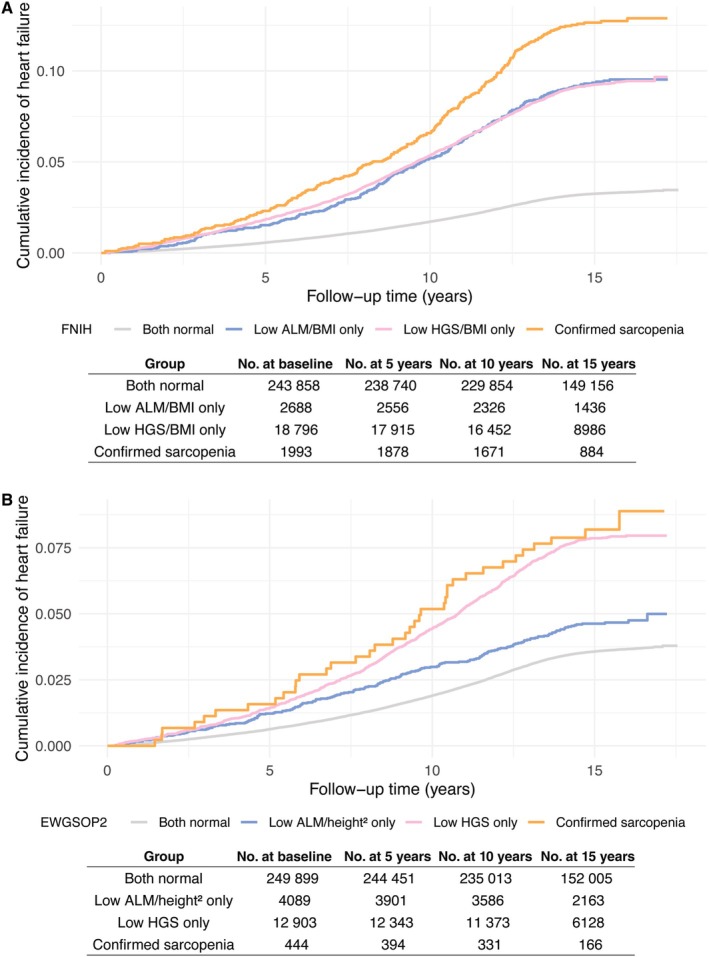
Cumulative incidence of heart failure across sarcopenia phenotypes defined by FNIH (A) and EWGSOP2 (B), estimated using the Aalen–Johansen method (death as competing risk). Risk set counts at baseline and at 5, 10, and 15 years are shown. ALM indicates appendicular lean mass; BMI, body mass index; EWGSOP2, European Working Group on Sarcopenia in Older People 2; FNIH, Foundation for the National Institutes of Health; and HGS, handgrip strength.

Using the FNIH definition, cumulative incidence curves showed the highest HF risk in the confirmed sarcopenia group, with lower risk in the low HGS/BMI only and low ALM/BMI only, whereas the both‐normal group maintained the lowest incidence (Figure 1A). Incidence rates per 1000 person‐years were 9.45, 6.76, and 6.69, respectively, compared with 2.22 in the reference group. In fully adjusted models, confirmed sarcopenia was associated with a 63% higher HF risk (HR, 1.63 [95% CI, 1.44–1.85]), the low HGS/BMI only group showed the largest increased risk (HR, 1.76 [95% CI, 1.66–1.85]), and the low ALM/BMI only group demonstrated a weaker but still significant association (HR, 1.20 [95% CI, 1.06–1.36]) (Table 2). Results were broadly consistent using the European Working Group on Sarcopenia in Older People 2 definition, with low HGS associated with higher HF risk (HR, 1.43, [95% CI, 1.34–1.53]), whereas low ALM/height^2^ was not (Figure 1B, Table 2). These findings were robust in sensitivity analyses using complete‐case data and after excluding events within the first 2 years (Tables S4 and S5).

**Table 2:** Association Between Sarcopenia Phenotypes and Risk of Incident HF

Group	No. (HF events/total)	Incidence rate (per 1000 person‐y)	HR (95% CI), Model 1	HR (95% CI), Model 2	HR (95% CI), Model 3
FNIH					
Both normal	7988/243 858	2.22	Reference	Reference	Reference
Low ALM/BMI only	253/2688	6.76	3.11 (2.74–3.52)‡	1.38 (1.21–1.56)‡	1.20 (1.06–1.36)†
Low HGS/BMI only	1739/18 796	6.69	3.04 (2.89–3.21)‡	2.24 (2.13–2.37)‡	1.76 (1.66–1.85)‡
Confirmed sarcopenia	253/1993	9.45	4.36 (3.84–4.94)‡	2.22 (1.96–2.52)‡	1.63 (1.44–1.85)‡
EWGSOP2					
Both normal	8994/249 899	2.44	Reference	Reference	Reference
Low ALM/height^2^ only	191/4089	3.33	1.38 (1.20–1.59)‡	0.80 (0.70–0.93)†	0.93 (0.81–1.08)
Low HGS only	1011/12 903	5.64	2.32 (2.18–2.48)‡	1.69 (1.58–1.80)‡	1.43 (1.34–1.53)‡
Confirmed sarcopenia	37/444	6.76	2.86 (2.07–3.95)‡	1.39 (1.01–1.93)*	1.37 (0.99–1.90)

Sarcopenia phenotypes were defined by FNIH and EWGSOP2 criteria. Model 1: unadjusted; Model 2: adjusted for age, sex, race, education, and deprivation; Model 3: further adjusted for protein intake, physical activity, smoking, alcohol use, diabetes, hypertension, coronary heart disease, and chronic kidney disease. ALM indicates appendicular lean mass; BMI body mass index; EWGSOP2, European Working Group on Sarcopenia in Older People 2; FNIH, Foundation for the National Institutes of Health; HGS, handgrip strength; HF, heart failure; and HR, hazard ratio.

* *P*<0.050.

^†^ *P*<0.01.

^‡^ *P*<0.001.

### Subgroup Analyses

**Figure 2:**
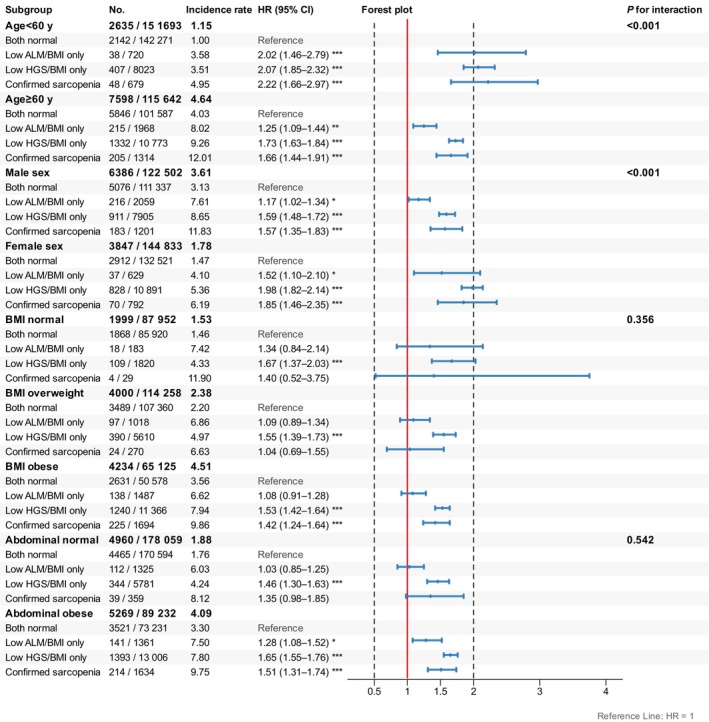
Subgroup analyses of sarcopenia phenotype and incident heart failure across age, sex, and adiposity strata (FNIH criteria). The covariate used to define the stratum was excluded from the full adjustment set to avoid overadjustment. *P* values indicate interaction across subgroups. ALM indicates appendicular lean mass; BMI, body mass index; FNIH, Foundation for the National Institutes of Health; HGS, handgrip strength; and HR, hazard ratio. * *P* < 0.050; ** *P* < 0.01; *** *P* < 0.001.

As shown in Figure 2, statistically significant interactions were observed between sarcopenia phenotypes and age or sex (both *P* for interaction <0.001) but not with BMI (*P*=0.356) or abdominal obesity status (*P*=0.542).

In participants aged <60 years, all FNIH‐defined sarcopenia phenotypes were associated with higher HF risk, with HRs of 2.02 (95% CI, 1.46–2.79) for low ALM/BMI, 2.07 (95% CI, 1.85–2.32) for low HGS/BMI, and 2.22 (95% CI, 1.66–2.97) for confirmed sarcopenia, whereas in those ≥60 years the associations were attenuated but remained significant, particularly for confirmed sarcopenia (HR, 1.66, 95% CI: 1.44–1.91) and low HGS/BMI (HR, 1.73, 95% CI: 1.63–1.84). Associations were also stronger in women than in men. Among women, the HRs for confirmed sarcopenia and low HGS/BMI only were 1.85 (95% CI, 1.46–2.35) and 1.98 (95% CI, 1.82–2.14), respectively, compared with 1.57 (95% CI, 1.35–1.83) and 1.59 (95% CI, 1.35–1.83) in men. Interaction‐adjusted marginal predictions showed similar patterns across sarcopenia phenotypes by age and sex (Figure S2). Confirmed sarcopenia was associated with higher HF risk in the obese (HR, 1.42 [95% CI, 1.24–1.64]) and abdominal‐obese (HR, 1.51 [95% CI, 1.31–1.74]) strata but not in groups without obesity, including BMI normal (HR, 1.40 [95% CI, 0.52–3.75]), overweight (HR, 1.04 [95% CI, 0.69–1.55]), or nonabdominal‐obese (HR, 1.35 [95% CI, 0.98–1.85]) strata. Notably, the low HGS/BMI only group showed the strongest and consistent associations with incident HF across all subgroups (all *P*<0.001).

Further, in magnetic resonance imaging subsets (n=732 within ±3 years and n=1254 within ±5 years of HF diagnosis), no significant associations were observed between sarcopenia indicators and left ventricular EF‐defined HF subtypes (Table S6).

### Associations Between Sarcopenia Indicators and Incident HF

**Figure 3:**
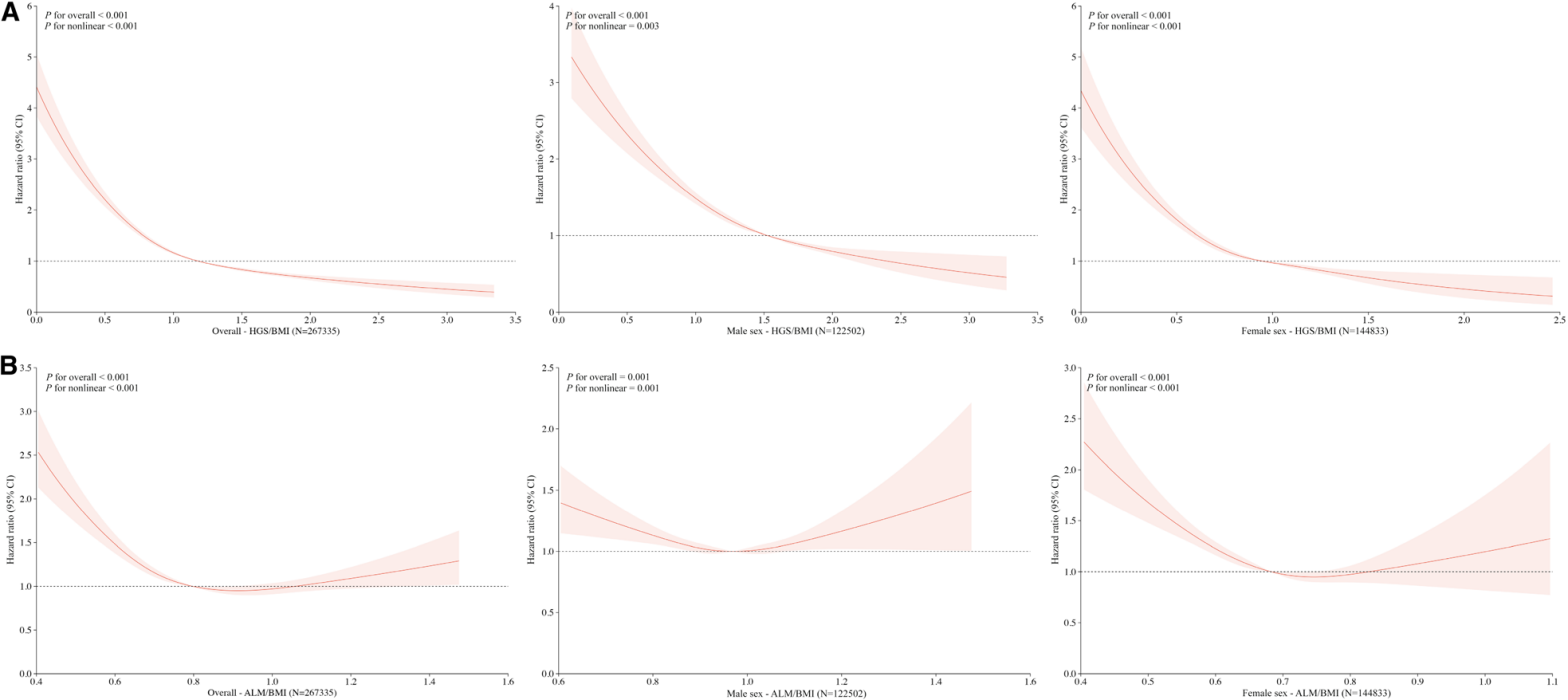
Dose–response relationships of HGS/BMI (A) and ALM/BMI (B) with incident heart failure in the overall population and by sex. ALM indicates appendicular lean mass; HGS, handgrip strength; and BMI, body mass index.

Both HGS/BMI and ALM/BMI exhibited significant nonlinear inverse associations with incident HF (*P* for overall <0.001; *P* for nonlinearity <0.001), showing L‐shaped relationships with the steepest risk increases at the lower ends of the distributions (Figure 3). HGS showed similar inverse trends, whereas ALM/height^2^ displayed a J‐shaped association (Figure S3).

Segmented models identified sex‐specific inflection points for HGS/BMI (1.48 in men, 0.82 in women) and ALM/BMI (0.85 in men, 0.72 in women). After comprehensive adjustment, each 1‐unit increase in HGS/BMI was strongly and inversely associated with incident HF (HR, 0.51 [95% CI, 0.47–0.55] in men; HR, 0.31 [95% CI, 0.27–0.36] in women; both *P*<0.001), whereas each one‐unit increase in ALM/BMI showed weaker or inconsistent effects (HR, 0.85 [95% CI, 0.62–1.17], *P*=0.32 in men; HR, 0.19 [95% CI, 0.12–0.30], *P*<0.001 in women). Subgroup analyses further demonstrated that reduced muscle strength (HGS/BMI and absolute HGS) was consistently associated with higher HF risk, whereas ALM/BMI showed weaker effects and ALM/height^2^ consistently inverse associations (Tables 3, Figures S4 and S5).

**Table 3:** Sex‐Specific Segmented Cox Regression of Sarcopenia Indicators (FNIH and EWGSOP2 Defined) With Incident Heart Failure

	Male sex		Female sex	
	No.	Adjusted HR (95% CI), *P* value	N	Adjusted HR (95% CI), *P* value
FNIH‐defined indicators				
HGS/BMI				
Total linear effect	122 502	0.51 (0.47–0.55)–<0.001	144 833	0.31 (0.27–0.36)–<0.001
Segmented Cox proportional hazards model				
Inflection point		1.48		0.82
Categorical effect				
Higher HGS/BMI	67 229	Ref	96 895	Ref
Lower HGS/BMI	55 273	1.44 (1.36–1.52)–<0.001	47 938	1.60 (1.50–1.72)–<0.001
*P* for log‐likelihood ratio		0.001		<0.001
ALM/BMI				
Total linear effect	122 502	0.85 (0.62–1.17)–0.320	144 833	0.19 (0.12–0.30)–<0.001
Segmented Cox proportional hazards model				
Inflection point		0.85		0.72
Categorical effect				
Higher ALM/BMI	109 180	Ref	48 851	Ref
Lower ALM/BMI	13 322	1.09 (1.03–1.17)–<0.01	95 982	1.21 (1.11–1.32)–<0.001
*P* for log‐likelihood ratio		<0.001		<0.001
EWGSOP2 defined indicators				
HGS				
Total linear effect	122 502	0.99 (0.98–0.99)–<0.001	144 833	0.97 (0.97–0.98)–<0.001
Segmented Cox proportional hazards model				
Inflection point		41		20
Categorical effect				
Higher HGS	65 576	Ref	121 740	Ref
Lower HGS	56 926	1.18 (1.12–1.25)–<0.001	23 093	1.42 (1.32–1.53)–<0.001
*P* for log‐likelihood ratio		0.004		<0.001
ALM/height^2^				
Total linear effect	122 502	1.28 (1.26–1.31)–<0.001	144 833	1.41 (1.36–1.45)–<0.001
Segmented Cox proportional hazards model				
Inflection point		8.18		6.45
Categorical effect				
Higher ALM/height^2^	83 695	Ref	103 930	Ref
Lower ALM/height^2^	38 807	0.75 (0.71–0.80)–<0.001	40 903	0.78 (0.72–0.85)–<0.001
*P* for log‐likelihood ratio		<0.001		<0.001

Sex‐specific inflection points were identified using the segmented package, and “Lower” vs “Higher” categories were defined by these thresholds. Models were adjusted for age, sex, race, education, Townsend deprivation index, protein intake, physical activity, smoking, alcohol use, diabetes, hypertension, coronary heart disease, and chronic kidney disease. ALM indicates appendicular lean mass; BMI, body mass index; EWGSOP2, European Working Group on Sarcopenia in Older People 2; FNIH, Foundation for the National Institutes of Health; HGS, handgrip strength; and HR, hazard ratio.

### Metabolomic Characterization of Sarcopenia Phenotypes and Risk of HF

*Limma* analysis identified multiple metabolites significantly different between the groups with confirmed sarcopenia and low HGS/BMI only versus reference group (Tables S7 and S8). Volcano plots (Figure S6a,b) illustrated distinct differential metabolite profiles across sarcopenia phenotypes. Pathway analysis showed enrichment of phenylalanine, tyrosine, and tryptophan biosynthesis and phenylalanine metabolism in both groups with confirmed sarcopenia and low HGS/BMI only, with linoleic acid metabolism additionally enriched in the latter (Figure S6c,d; Tables S9 and S10). In repeat‐assessment analyses, differential metabolite patterns between the groups with confirmed sarcopenia and low HGS/BMI only versus the reference group (Tables S11 and S12) were largely consistent with the primary findings, supporting the reproducibility of the observed metabolic signatures.

Cox models identified multiple features significantly associated with incident HF in both the groups with confirmed sarcopenia and low HGS/BMI (Table 4, Tables S13 and S14). Shared associations across both phenotypes included higher levels of glycoprotein acetyls and glucose–lactate, and lower omega‐3 fatty acids, docosahexaenoic acid, glycine, glutamine, and histidine, all associated with higher HF risk. Distinct associations included elevated 3‐hydroxybutyrate in the confirmed sarcopenia group, and higher citrate with lower leucine in the group with low HGS/BMI, all associated with increased HF risk. Pearson correlation heatmaps were generated for metabolites meeting the predefined significance thresholds in each phenotype group (Figure S7).

**Table 4:** Significant Associations Between Differential Metabolites (Adjusted *P*<0.01) and Incident Heart Failure Among Participants With Low HGS/BMI Only Versus Reference Groups

Metabolite	Category	HR (95% CI)	Adjusted *P* value
Tyrosine	Amino acids	1.07 (1.05–1.10)	4.23E‐11
Phenylalanine		1.05 (1.04–1.07)	3.31E‐08
Leucine		0.96 (0.94–0.98)	0.019
Glycine		0.93 (0.91–0.96)	1.70E‐06
Glutamine		0.96 (0.94–0.98)	0.005
Histidine		0.91 (0.89–0.93)	3.92E‐16
Creatinine		1.06 (1.04–1.08)	3.47E‐08
Glucose‐lactate	Carbohydrates (glycolysis/tricarboxylic acid)	1.08 (1.06–1.10)	8.44E‐14
Glucose		1.08 (1.06–1.10)	1.65E‐12
Citrate		1.05 (1.03–1.07)	6.85E‐05
Docosahexaenoic acid	Fatty acids	0.89 (0.87–0.91)	2.98E‐23
Omega‐3 fatty acids		0.90 (0.88–0.92)	1.38E‐20
Linoleic acid		0.95 (0.93–0.97)	1.07E‐05
Polyunsaturated fatty acids		0.93 (0.91–0.95)	1.71E‐09
Albumin	Proteins others/fluid balance	0.92 (0.91–0.93)	1.24E‐55
Glycoprotein acetyls		1.13 (1.11–1.15)	4.77E‐28
LDL‐cholesterol	Cholesterol	0.94 (0.92–0.96)	1.07E‐07
Clinical LDL‐cholesterol		0.95 (0.93–0.96)	8.71E‐06
Cholesterol in large LDL		0.94 (0.92–0.96)	4.26E‐07
Cholesterol in medium LDL		0.94 (0.92–0.96)	9.90E‐08
Total cholesterol		0.95 (0.93–0.97)	0.001
CE in small HDL	Cholesteryl esters	0.90 (0.88–0.91)	1.22E‐22
CE in large LDL		0.94 (0.92–0.96)	2.29E‐07
CE in medium LDL		0.94 (0.92–0.96)	2.49E‐06
CE in HDL		0.95 (0.93–0.98)	0.011
CE in medium HDL		0.95 (0.93–0.97)	0.001
CE in small LDL		0.95 (0.93–0.97)	4.34E‐04
FC in medium LDL	Free cholesterol	0.93 (0.91–0.95)	1.33E‐09
FC in small LDL		0.94 (0.92–0.96)	1.26E‐07
FC in LDL		0.94 (0.92–0.96)	4.36E‐07
FC in very large HDL		1.07 (1.05–1.09)	2.49E‐07
FC in large LDL		0.94 (0.92–0.96)	1.52E‐05
FC in very small VLDL		1.06 (1.04–1.08)	1.27E‐05
Triglycerides in intermediate‐density lipoprotein	Triglycerides	1.08 (1.05–1.10)	6.99E‐11
Triglycerides in large LDL		1.06 (1.04–1.09)	4.06E‐08
Triglycerides in very small VLDL		1.06 (1.04–1.08)	8.38E‐06
Triglycerides in LDL		1.05 (1.03–1.07)	5.59E‐05
Triglycerides in large HDL		1.05 (1.03–1.08)	1.06E‐04
Triglycerides in very large HDL		1.05 (1.03–1.07)	7.35E‐04
Triglycerides in medium LDL		1.04 (1.02–1.06)	0.033
Triglycerides in medium VLDL		0.96 (0.94–0.98)	0.026
Phospholipids in very small VLDL	Phospholipids	1.07 (1.05–1.09)	3.20E‐09
Phospholipids in small HDL		0.95 (0.93–0.97)	5.92E‐05
Phospholipids in very large HDL		1.06 (1.03–1.08)	2.45E‐04
Phospholipids in large LDL		0.95 (0.93–0.97)	4.00E‐04
Phospholipids in medium LDL		0.94 (0.92–0.96)	1.45E‐06
Total lipoprotein particle concentration	Lipoprotein size/particles	0.93 (0.91–0.95)	2.25E‐07

Hazard ratios and 95% CIs represent the risk of incident heart failure per 1‐SD increase in log_10_‐transformed, standardized metabolite concentrations.

BMI indicates body mass index; CE, cholesteryl esters; DHA; FC, free cholesterol; HDL, high‐density lipoprotein; HGS, handgrip strength; HR, hazard ratio; LDL, low‐density lipoprotein; and VLDL, very low‐density lipoprotein.

### Incremental Predictive Value and Mediation Effects of LASSO‐Selected Metabolites

LASSO selected metabolites are listed in Table S15. Incorporating selected metabolites into traditional risk factors model (base model) significantly improved HF risk prediction. For confirmed sarcopenia, incorporating 13 metabolites increased the C‐index from 0.792 to 0.802, with a 15‐year continuous NRI of 14.1% (95% CI, 12.8–15.1) and a corresponding IDI improvement of 1.0% (95% CI, 0.8–1.1). For the group with low HGS/BMI only, 14 metabolites increased the C‐index from 0.792 to 0.803, with a 15‐year NRI of 14.8% (95% CI, 13.7–16.1) and an IDI of 0.9% (95% CI, 0.8–1.0). Notably, adding HGS/BMI alone to the base model also improved prediction (C‐index 0.795; 15‐year NRI 5.2%; IDI 0.4%) (Table 5). Calibration remained good after addition of the metabolite panels or HGS/BMI, with close agreement between observed and predicted 15‐year HF risks across deciles and cross‐validated calibration slopes ranging from 0.995 to 0.998 (Figure S8).

**Table 5:** Incremental Predictive Value of Selected Metabolites and HGS/BMI for Incident Heart Failure

Group	15‐y continuous NRI, %	15‐y IDI, %	C‐index (95% CI)
Base model	Reference	Reference	0.792 (0.788–0.796)
|log_2_fold change| >0.02			
Base model +6 metabolites	12.0 (10.7–12.9)***	0.6 (0.5–0.7)***	0.798 (0.794–0.802)
Base model +4 metabolites	10.4 (9.3–11.4)***	0.5 (0.4–0.6)***	0.797 (0.793–0.801)
False discovery rate <0.01			
Base model +13 metabolites	14.1 (12.8–15.1)***	1.0 (0.8–1.1)***	0.802 (0.798–0.806)
Base model +14 metabolites	14.8 (13.7–16.1)***	0.9 (0.8–1.0)***	0.803 (0.799–0.807)
Base model + HGS/BMI (low vs normal)	5.2 (3.4–6.7)***	0.4 (0.3–0.5)***	0.795 (0.791–0.800)

The 15‐year continuous NRI (%) and IDI (%) were calculated by comparing models with and without selected metabolites using Cox‐based risk prediction. The base model included the full set of covariates (model 3 as in Table 2). BMI, body mass index; HGS, handgrip strength; IDI, integrated discrimination improvement; and NRI, net reclassification improvement.

*** *P*<0.001.

LASSO‐selected metabolites are all significant mediators in the relationship between sarcopenia phenotypes and incident HF (Table S16). In the group with confirmed sarcopenia, the proportion mediated ranged from 0.9% to 14.5%, with glycoprotein acetyls showing the strongest mediation effect. In the group with low HGS/BMI, mediation effects were generally smaller (0.3%–8.6%) but also observed across metabolites representing inflammatory, lipid, and amino acid pathways.

## DISCUSSION

In this large prospective study, FNIH‐defined sarcopenia and low HGS (absolute or relative to BMI) were independently associated with higher HF risk, with HGS proving a stronger predictor than muscle mass and associations being more pronounced in younger adults (<60 years) and women. Common metabolic signatures associated with both groups with confirmed sarcopenia and low HGS/BMI were higher glycoprotein acetyls, glucose–lactate, phenylalanine, and tyrosine and lower omega‐3 fatty acids, histidine, glycine, and glutamine. Further, participants with confirmed sarcopenia had elevated 3‐hydroxybutyrate, whereas the group with low HGS/BMI only was marked by lower leucine and higher citrate plasma concentration. Incorporating selected metabolites into traditional HF risk factors improved model discrimination and reclassification and partially mediated the associations.

### Clinical Associations Between Sarcopenia Phenotypes and Incident HF

FNIH‐defined confirmed sarcopenia was generally associated with higher HF risk, with significant associations observed in the overall cohort and across most subgroups, but not in participants without obesity (by BMI or waist circumference). This aligns with evidence that sarcopenic obesity confers greater cardiovascular disease risk than sarcopenia without obesity,^23^ likely due to its stronger association with insulin resistance, dyslipidemia, and metabolic syndrome.^24^, ^25^, ^26^ In contrast, the European Working Group on Sarcopenia in Older People 2‐defined confirmed sarcopenia was not associated with higher HF risk, as including ALM/height^2^ appeared to attenuate the effect. This discrepancy highlights that BMI‐adjusted muscle mass demonstrates superior predictive value for HF risk compared with height‐adjusted muscle mass, as the latter may overlook body shape variation. Prior study^27^ also supports ALM/BMI was superior to ALM/height^2^ for identifying muscle dysfunction in HF. This divergence likely reflects differences in the biological construct captured by the 2 definitions, where ALM/BMI captures relative muscle deficiency under metabolic load, better accounts for excess adiposity, and correlates more strongly with muscle strength, physical performance and metabolic risk, whereas ALM/height^2^ reflects stature‐adjusted lean mass alone and is more commonly linked to biomechanical outcomes such as falls and fractures.^28^ However, although BMI is a useful screening tool for sarcopenic obesity in younger adults,^29^ its value in older adults is limited, as adiposity often increases and muscle declines without major BMI changes.^9^, ^30^ Alternative adiposity measures such as waist‐to‐height ratio,^31^ waist circumference and regional fat mass may improve risk stratification, though standardized thresholds remain lacking.

Significant association between HGS and HF has also been reported in UKB and a US community cohort with <5 years follow‐up.^10^, ^11^ In contrast, ALM showed weaker or even inverse associations in our study, supporting prior evidence that muscle strength exerts a greater impact on cardiovascular disease risk than muscle mass.^32^

Age‐ and sex‐stratified analyses showed that all sarcopenia phenotypes were associated with higher HF risk compared with the reference group but more pronounced in adults <60 years and in women. The age‐specific patterns should be interpreted with caution, as UKB participants (baseline age 37–73 years) tend to develop HF earlier than the general population, which may overrepresent early‐onset cases. Stronger associations in younger adults may also reflect fewer competing comorbidities, whereas in older adults, longer prior exposure to sarcopenia before baseline and greater comorbidity burden may attenuate the observed risk. In women, lower baseline muscle reserves, hormonal profile,^33^ and sex‐specific patterns of adiposity^34^, ^35^ patterns may amplify the detrimental cardiovascular consequences of sarcopenia.

### Metabolic Signatures Underlying the Association of Sarcopenia and HF

Sarcopenia and HF share mechanisms including chronic low‐grade inflammation, insulin resistance, oxidative stress, and mitochondrial dysfunction,^8^, ^36^ many of which were reflected in our metabolite profiles.

Shared associations across the groups with confirmed sarcopenia and low HGS/BMI only included higher glycoprotein acetylation, a composite marker of circulating inflammatory cytokines,^37^ lower omega‐3 fatty acids (including docosahexaenoic acid) reflecting impaired anti‐inflammatory and antioxidative capacity,^38^, ^39^ and triglyceride enrichment in lipoprotein fractions may contribute to myocardial lipotoxicity,^40^ a process implicated in HF pathogenesis. Higher aromatic amino acids (phenylalanine and tyrosine), together with lower histidine, glycine, and glutamine concentrations pointed to skeletal muscle disrupted protein turnover, insulin resistance, and oxidative stress.^41^, ^42^ Several of these alterations have been linked to both sarcopenia and adverse HF outcomes.^43^, ^44^, ^45^, ^46^ Higher glucose–lactate further supports mitochondrial dysfunction and less efficient energy production.^47^

The group with low HGS/BMI exhibited additional significantly lower leucine and higher citrate. Lower leucine, more closely link to muscle strength rather than muscle mass,^48^ likely reflects altered branched‐chain amino acid metabolism and reduced availability for anabolic signaling, particularly mTORC1 (mechanistic target of rapamycin complex 1) activation,^49^ which is essential for protein synthesis and cellular growth. Impaired mTORC1 signaling may contribute to anabolic resistance, muscle catabolism, and, ultimately, cardiac dysfunction.^50^ Higher citrate indicates tricarboxylic acid cycle dysregulation, consistent with prior observations of increased tricarboxylic acid intermediates in HF.^51^ Interestingly, Selvaraj et al.^52^ also reported that elevated metabolites indicative of mitochondrial dysfunction and aromatic amino acids, together with reduced branched‐chain amino acids, were associated with adverse HF outcomes, hypothesizing that such systemic alterations may originate from peripheral tissues such as skeletal muscle. Our findings further support this interpretation. Notably, 3‐hydroxybutyrate was uniquely higher in confirmed sarcopenia. As a ketone metabolite, it has been proposed as a cardioprotective fuel,^51^, ^53^ yet has also been found elevated in plasma and myocardial tissue of patients with HF with reduced EF but not HF with preserved EF, suggesting subtype heterogeneity. Although the mechanism remain uncertain, these findings highlight the need to examine sarcopenia in conjunction with HF subtypes, as muscle mass and function may substantially influence systemic metabolism.^54^, ^55^

Notably, the improved risk discrimination metrics (NRI and IDI) from selected metabolites further supports their potential as early biomarkers for HF. Mediation analyses further suggested that systemic metabolic alterations may partially mediated the association between sarcopenia phenotypes and higher HF risk. Collectively, these findings underscore that sarcopenia‐related metabolic signatures capture systemic pathophysiological processes implicated in HF development.

### Implications

These findings have several clinical and translational implications. First, routine screening for FNIH‐defined sarcopenia or with a simple, reproducible HGS assessment would facilitate HF risk stratification in general population. Second, circulating metabolite profiles provide minimally invasive tools to identify higher‐risk individuals and inform tailored lifestyle and muscle‐centric preventive strategies. Third, integrating HGS and metabolic information into HF risk models could enhance precision prevention strategies. Moreover, our analyses implicate lipid, amino acid, and energy metabolism pathways as potential targets linking muscle dysfunction to the development of HF.

### Strengths and Limitations

Key strengths of this study include its large sample size, prospective design, objective skeletal muscle phenotyping using two international definitions of sarcopenia, integration of clinical and metabolomic data, and long follow‐up. However, certain limitations should be acknowledged. First, muscle mass was estimated using bioelectrical impedance analysis rather than dual‐energy X‐ray absorptiometry, although validated equations were applied. Second, all muscle and metabolite measurements were mainly taken at baseline, precluding assessment of longitudinal changes. Third, HF subtypes (HF with reduced EF and HF with preserved EF) were unavailable cohort‐wide and only be approximated in a small magnetic resonance imaging‐left ventricular EF subset. Fourth, the UKB's “healthy volunteer” bias and relatively young baseline age range may limit generalizability, particularly to the oldest old. Fifth, our risk prediction analyses were conducted within UKB but not externally validated, although robustness was supported by 1000 bootstrap resamples. Sixth, metabolomic profiling was restricted to the NMR platform, which lacks the broader coverage of mass spectrometry. Finally, the observational design precludes causal inference, and reverse causation cannot be fully excluded, though sensitivity analyses excluding early HF cases yielded similar results. The identified metabolic candidates potentially linking sarcopenia to HF also require confirmation in mechanistic or interventional studies, and residual confounding remains possible.

## CONCLUSIONS

FNIH‐defined sarcopenia, particularly low HGS, was independently associated with an increased HF risk in this large prospective cohort, with stronger associations observed in younger adults and women. Circulating metabolites reflecting inflammation, lipid, amino acid, and energy metabolism were associated with sarcopenia‐related states and prospectively linked to HF. Incorporating these metabolites modestly improved risk discrimination beyond traditional cardiovascular risk factors, with evidence of partial mediation, supporting their potential as early biomarkers for HF risk stratification.

## Sources of Funding

Ziyi Zhong is supported by China Scholarship Council‐University of Liverpool joint scholarship.

## Disclosures

None.

## Supporting information

Tables S1–S16
Figures S1–S8
Supplemental Methods
References 56–60

STROBE Statement
