# Supplementary material for: Sarcopenia and Its Associated Metabolic Profile Predict Incident Heart Failure: A Prospective Cohort Study of 267 335 Adults in the UK Biobank
Source: J Am Heart Assoc. 2026 Jul 17;15(14):e047621. doi: 10.1161/JAHA.125.047621 (PMC13477333; doi:10.1161/JAHA.125.047621)
Supplement: Supplementary file 1 [file JAH3-15-e047621-s001.pdf]

Supplemental Material

Supplemental Methods

Data S1. Assessment and Cut-Off Points of Sarcopenia

HGS was assessed using a Jamar J00105 hydraulic hand dynamometer; the maximum value from either hand was recorded and normalized by BMI (HGS/BMI ratio). Muscle mass was assessed using BIA, from which appendicular lean mass (ALM) was derived and normalized by BMI (ALM/BMI ratio) using an established and widely used equation: ALM (kg) = 0.958 × appendicular fat-free mass (kg) − 0.166 × sex − 0.308, where sex = 1 for male and 0 for female[56].

Sarcopenia was defined by two established criteria:1) Foundation for the National Institutes of Health (FNIH): low HGS/BMI (<1.00 for males, <0.56 for females) and low ALM/BMI (<0.789 for males, <0.512 for females)[19]; 2) European Working Group on Sarcopenia in Older People 2 (EWGSOP2): low HGS (<27 kg for males, <16 kg for females) and low ALM/height² (<7.0 kg/m² for males, <5.5 kg/m² for females)[7].

Data S2. Plasma biomarker profiling by NMR

Metabolic profiling was performed on baseline plasma samples using a high-throughput proton NMR spectroscopy platform developed by Nightingale Health Ltd (Helsinki, Finland). The NMR measurements were carried out between 2019 and 2020 using six 500 MHz NMR spectrometers (Bruker AVANCE IIIHD), following a standardized and quality-controlled protocol. Plasma samples were stored at −80 °C prior to analysis. Each sample was thawed at 4 °C overnight, centrifuged, and mixed with phosphate buffer before being transferred into NMR tubes. Quality assurance procedures included two blinded duplicate samples and two internal quality control samples on each 96-well plate to monitor analytical consistency across batches and spectrometers. The coefficients of variation were <5% for the majority of biomarkers. Technical reproducibility was further validated using repeat measurements from longitudinal samples taken 4 years apart, showing high concordance across time points and instruments.

All biomarker values were derived using proprietary quantification algorithms by Nightingale Health and subjected to rigorous calibration and validation procedures. The profiling covered 249 metabolic features, including routine lipids, lipoprotein subclasses, fatty acids, amino acids, glycolysis-related metabolites, ketone bodies, and other low-molecular-weight compounds.

Data S3. Multiple imputation procedure

Missing covariate data were imputed using multiple imputation by chained equations (MICE) in R. Five imputed datasets were generated with 20 iterations each. Predictive mean matching was used for continuous variables, logistic regression for binary variables, and proportional odds logistic regression for ordered categorical variables. Diagnostics (e.g., density plots and strip plots) confirmed consistency between imputed and observed values. All cox models were fitted within each imputed dataset, and pooled estimates were obtained using Rubin’s rules via the with() and pool() functions. For non-survival analyses, the first completed dataset was used.

Data S4. Metabolomics analyses

Differential metabolite analysis was performed using covariate-adjusted linear models implemented in the *limma* R package, with variance moderation achieved through the empirical Bayes (eBayes) method[57]. Two thresholds were applied to define significant alterations: (1) false discovery rate (FDR) <0.01 and (2) absolute $\log_2$ fold change greater than 0.02 with FDR <0.05. Lipoprotein structural variables were excluded from downstream analyses to reduce redundancy.

Pathway enrichment analysis was performed using MetaboAnalyst 6.0[58], mapping metabolites to Kyoto Encyclopedia of Genes and Genomes (KEGG) pathways. Pathways were considered biologically relevant if they met an FDR threshold of <0.05 and an impact score greater than 0.2[59].

Cox proportional hazards models were used to examine associations between individual metabolites and incident HF. Metabolites reaching Bonferroni-corrected significance were subsequently entered into a least absolute shrinkage and selection operator (LASSO) Cox model using the *glmnet* package to identify a parsimonious predictor set.

Incremental predictive performance beyond conventional risk factors was assessed by calculating Harrell's C-index (*Hmisc* package), continuous net reclassification improvement (NRI), and integrated discrimination improvement (IDI). These analyses were implemented using the *survIDINRI* package, with 1,000 bootstrap resamples applied to derive 95% confidence intervals and P-values.

All metabolite concentrations were $\log_2$-transformed prior to differential expression analysis. For Cox and prediction models, metabolites were $\log_{10}$-transformed and standardized to z-scores.

Table S1. Definitions and assessments of variables.

Covariates	UK Biobank Field ID	ICD-10 Code or Definition
Age	21022	/
Sex	31	
BMI	21001	
Waist circumference	48	
Ethnicity	21000	
education level	6138	
Townsend deprivation index	189	
Numbers of Total weekly protein servings	1329 oily fish, 1339 non-oily fish, 1349 processed meat intake, 1359 poultry, 1369 beef, 1379 lamb, 1389 pork	
Summed MET mins/week for PA	22040	
Smoking status	20116	
Alcohol Frequency	20117	
Diabetes	130706, 130708, 130710, 130712, 130714	• E10 (Type 1 diabetes mellitus)<br>• E11(Type 2 diabetes mellitus)<br>• E12 (malnutrition-related diabetes mellitus)<br>• E13 (Other specified diabetes mellitus)<br>• E14 (Unspecified diabetes mellitus)
Hypertension	131286,131288,131290,131292, 131294, 132180	• I10 (essential [primary] hypertension)<br>• I11 (hypertensive heart disease)<br>• I12 (hypertensive renal disease)<br>• I13 (hypertensive heart and renal disease)<br>• I15 (secondary hypertension)<br>• O10 (pre-existing hypertension complicating pregnancy)
CHD	131296,131298, 131300, 131302, 131304, 131306	• I20 (angina pectoris)<br>• I21 (acute myocardial infarction)<br>• I22 (subsequent myocardial infarction)<br>• I23 (complications following acute MI)<br>• I24 (other acute ischaemic heart diseases)<br>• I25 (chronic ischaemic heart disease)
CKD	30700 Creatinine	CKD was identified using estimated glomerular filtration rate (eGFR) values calculated from serum creatinine measured at the UK Biobank baseline assessment. The eGFR was derived using the Chronic Kidney Disease Epidemiology Collaboration (CKD-EPI) equation[60]. Participants with eGFR values below 60 mL/min/1.73 m² were classified as having CKD, while those with values at or above this threshold were considered non-CKD.

Abbreviations: BMI, body mass index; MET, Metabolic Equivalent of Task; Mins, minutes; CHD, Coronary Heart Disease; CKD, Chronic Kidney Disease

Table S2. Covariates missingness summary (total N=267,335)

Variable	Missing, N	Missing %
Summed MET mins/week for physical activity	60643	22.7%
CKD	11798	4.41%
Smoking Status	1255	0.5%
Alcohol Frequency	513	0.2%
Townsend deprivation index	324	0.1%
Waist circumference	44	0.02%
All the rest (Age, Sex, Ethnicity, Education, Numbers of Total weekly protein servings, etc.)	0	0%

Abbreviations: MET, Metabolic Equivalent of Task; Mins, minutes; BMI, body mass index.

Table S3. Baseline Characteristics of Participants by Heart Failure Incidence Status

	**Overall (N= 267, 335)**	**No HF (N= 257, 102)**	**New-onset HF (N= 10, 233)**	***P* value**
**Sociodemographic Characteristics**				
Age, years	56.52 (8.08)	56.30 (8.07)	62.08 (6.15)	<0.001
Sex, Male, n (%)	122,502 (45.8)	116,116 (45.2)	6,386 (62.4)	<0.001
Ethnicity, White, n (%)	252,898 (94.6)	243,158 (94.6)	9,740 (95.2)	0.008
Education, College or University degree (%)	84,905 (31.8)	82,858 (32.2)	2,047 (20.0)	<0.001
Townsend Deprivation Index	-1.37 (3.07)	-1.39 (3.05)	-0.76 (3.32)	<0.001
**Clinical Measurements**				
BMI (kg/m²)	27.43 (4.76)	27.34 (4.69)	29.73 (5.74)	<0.001
BMI group				<0.001
BMI normal (%)	87,952 (32.9)	85,953 (33.4)	1,999 (19.5)	
BMI overweight (%)	114,258 (42.7)	110,258 (42.9)	4,000 (39.1)	
BMI obesity (%)	65125 (24.4)	60,891 (23.7)	4,234 (41.4)	
Waist circumference, cm	90.20 (13.40)	89.87 (13.23)	98.51 (14.75)	<0.001
Waist circumference group				<0.001
Non-abdominal obesity (%)	178,059 (66.6)	173,099 (67.3)	4,960 (48.5)	
Abdominal obese (%)	89,232 (33.4)	83,963 (32.7)	5,269 (51.5)	
**Lifestyle Factors**				
Numbers of Total weekly protein servings	3.70 (1.67)	3.70 (1.67)	3.78 (1.68)	<0.001
Summed MET mins/week for PA	2,677.07 (2675.47)	2,680.19 (2671.66)	2,598.60 (2768.41)	0.002
Smoking Status (%)				<0.001
Current	28,157 (10.5)	26,548 (10.3)	1,609 (15.7)	
Previous	92,778 (34.7)	88,265 (34.3)	4,513 (44.1)	
Never	146,400 (54.8)	142,289 (55.3)	4,111 (40.2)	
Alcohol Frequency (%)				<0.001
Daily or almost daily	53,958 (20.2)	51,712 (20.1)	2,246 (21.9)	
Three or four times a week	62,336 (23.3)	60,328 (23.5)	2,008 (19.6)	
Once or twice a week	69,910 (26.2)	67,591 (26.3)	2,319 (22.7)	
One to three times a month	29,863 (11.2)	28,829 (11.2)	1,034 (10.1)	
Special occasions only	30,361 (11.4)	28,961 (11.3)	1,400 (13.7)	
Never	20,907 (7.8)	19,681 (7.7)	1,226 (12.0)	
**Comorbidities**				
Diabetes (%)	13,561 (5.1)	11,867 (4.6)	1,694 (16.6)	<0.001
Hypertension (%)	71,379 (26.7)	65,968 (25.7)	5,411 (52.9)	<0.001
CHD (%)	13,555 (5.1)	11,285 (4.4)	2,270 (22.2)	<0.001
CKD (%)	6,044 (2.3)	5,287 (2.1)	757 (7.4)	<0.001
**HGS/BMI FNIH group (%)**				<0.001
low	20,789 (7.8)	18,797 (7.3)	1,992 (19.5)	
normal	246,546 (92.2)	238,305 (92.7)	8,241 (80.5)	
**ALMBMI/FNIH group (%)**				<0.001
low	4,681 (1.8)	4,175 (1.6)	506 (4.9)	
normal	262,654 (98.2)	252,927 (98.4)	9,727 (95.1)	

Data were shown as mean (standard deviation), or n (%).

Abbreviations: HGS, handgrip strength; HF, heart failure; BMI, body mass index; MET, Metabolic Equivalent of Task; Mins, minutes; CHD, Coronary Heart Disease; CKD, Chronic Kidney Disease

Table S4. Association Between Combined Sarcopenia Phenotypes and Risk of Incident Heart Failure (unimputed, N=196, 835)

Group	N (HF Events / Total)	Incidence rate (per 1000 PY)	HR (95% CI), Model 1	HR (95% CI), Model 2	HR (95% CI), Model 3
Both normal	5,621 / 181,770	2.09	Reference	Reference	Reference
Low ALM/BMI only	156 / 1,769	6.32	3.08 (2.63–3.61) ***	1.34 (1.14–1.58) ***	1.15 (0.98–1.35)
Low HGS/BMI only	1,095 / 12,146	6.5	3.13 (2.94–3.34) ***	2.29 (2.14–2.44) ***	1.76 (1.65–1.89) ***
Confirmed sarcopenia	139 / 1,150	8.96	4.38 (3.70–5.19) ***	2.16 (1.83–2.57) ***	1.56 (1.32–1.85) ***

Incidence rates were calculated as the number of events per 1,000 person-years. Model 1: unadjusted; Model 2: adjusted for age, sex, ethnicity, education, and deprivation; Model 3: further adjusted for protein intake, physical activity, smoking, alcohol use, diabetes, hypertension, coronary heart disease, and chronic kidney disease.
Abbreviations: BMI, body mass index; ALM, appendicular lean mass; HGS, handgrip strength; PY, person-years; FNIH, Foundation for the National Institutes of Health; EWGSOP2, European Working Group on Sarcopenia in Older People 2.

Table S5. Association Between Combined Sarcopenia Phenotypes and Risk of Incident Heart Failure (exclude 2 years, N=266, 795)

Group	N (HF Events / Total)	Incidence rate (per 1000 PY)	HR (95% CI), Model 1	HR (95% CI), Model 2	HR (95% CI), Model 3
**FNIH**					
Both normal	7585 / 243455	2.11	Reference	Reference	Reference
Low ALM/BMI only	243 / 2678	6.5	3.16 (2.78–3.59) ***	1.41 (1.24–1.60) ***	1.23 (1.08–1.40) **
Low HGS/BMI only	1627 / 18684	6.27	3.01 (2.85–3.18) ***	2.22 (2.10–2.34) ***	1.75 (1.65–1.85) ***
Confirmed sarcopenia	238 / 1978	8.9	4.34 (3.82–4.94) ***	2.22 (1.95–2.53) ***	1.64 (1.44–1.87) ***
**EWGSOP2**					
Both normal	8532 / 249437	2.32	Reference	Reference	Reference
Low ALM/height^2 only	175 / 4073	3.05	1.34 (1.15–1.55) ***	0.78 (0.67–0.91) **	0.91 (0.78–1.06)
Low HGS only	952 / 12844	5.32	2.32 (2.17–2.48) ***	1.68 (1.57–1.80) ***	1.43 (1.34–1.54) ***
Confirmed sarcopenia	34 / 441	6.22	2.81 (2.00–3.93) ***	1.38 (0.98–1.93)	1.37 (0.97–1.92)

Sarcopenia phenotypes were defined according to the FNIH and EWGSOP2 criteria. Incidence rates were calculated as the number of events per 1,000 person-years. Model 1 represents unadjusted Cox regression estimates. Model 2 includes adjustment for age, sex, ethnicity, education level, socioeconomic deprivation. Model 3 includes adjustment for age, sex, ethnicity, education level, Townsend deprivation index, protein intake, physical activity, smoking, alcohol use, diabetes, hypertension, coronary heart disease, and chronic kidney disease. Hazard ratios (HRs) and 95% confidence intervals (CIs) are shown relative to the reference group.
Abbreviations: BMI, body mass index; ALM, appendicular lean mass; HGS, handgrip strength; PY, person-years; FNIH, Foundation for the National Institutes of Health; EWGSOP2, European Working Group on Sarcopenia in Older People 2.

Table S6. Associations Between Sarcopenia Parameters and Heart Failure Subtypes (LVEF < 40% vs ≥50%) Defined by LVEF

	3-year Window, N (LVEF < 40%/≥50%)	OR (95% CI), Model 1	OR (95% CI), Model 2	5-year Window, N (LVEF < 40%/≥50%)	OR (95% CI), Model 1	OR (95% CI), Model 2
HGS/BMI	228/504	2.32 (0.87–6.19)	1.37 (0.37–5.17)	366/888	2.12 (1.01–4.46) *	1.03 (0.40–2.69)
ALM/BMI		26.80 (2.41–298.18) **	66.41 (0.93–4721.04)		30.85 (4.26–223.32) ***	13.57 (0.57–321.67)
HGS		1.03 (0.99–1.06)	0.99 (0.94–1.05)		1.03 (1.00–1.06)	0.99 (0.95–1.03)
ALM/height^2		1.03 (0.77–1.38)	0.78 (0.51–1.19)		1.12 (0.90–1.40)	0.91 (0.68–1.21)

OR and 95% CI for the association between sarcopenia parameters and heart failure subtypes (LVEF < 40% and ≥ 50%).

Model 1: unadjusted; Model 2: adjusted for age, sex, and ethnicity.

MRI measurements were within ±5 years or ±3 years of HF diagnosis.

* $P < 0.05$; ** $P < 0.01$; *** $P < 0.001$.

Abbreviations: LVEF, left ventricular ejection fraction; OR, odds ratios; CI, confidence intervals.

Table S7. Significant differential metabolites (FDR adjusted *P* value < 0.05) between Confirmed Sarcopenia(N=1,993) and Both Normal(N=243,858) (reference).

**Metabolite**	**Log2FC**	***P* Value**	**adj. *P* Value**	**B**
Monounsaturated Fatty Acids	0.0690604	1.29E-25	1.55E-24	44.2437538
Lactate	0.05214767	1.19E-12	5.41E-12	14.765481
Total Lipids in Large HDL	-0.0508712	4.68E-23	4.37E-22	38.4041161
Albumin	-0.0487634	3.37E-54	2.83E-52	109.669191
Total Triglycerides	0.04672041	3.77E-10	1.32E-09	9.12920324
Triglycerides in VLDL	0.04626221	6.02E-10	2.02E-09	8.67240716
Glycoprotein Acetyls	0.04519724	3.56E-107	5.97E-105	231.304342
Total Lipids in Chylomicrons and Extremely Large VLDL	0.0416292	1.74E-18	1.33E-17	27.9962675
Total Lipids in VLDL	0.03987519	3.67E-06	8.56E-06	0.23044969
Glucose-lactate	0.03955666	4.13E-16	2.39E-15	22.6024624
Saturated Fatty Acids	0.03869418	7.21E-11	2.82E-10	10.7452663
Cholesterol in Large HDL	-0.0373947	4.55E-27	5.89E-26	47.5571061
Omega-3 Fatty Acids	-0.0373464	5.20E-18	3.49E-17	26.9166153
Cholesteryl Esters in Large HDL	-0.0313877	1.16E-27	1.94E-26	48.9172963
Total Fatty Acids	0.03114436	2.09E-07	5.85E-07	2.98896393
Triglycerides in Chylomicrons and Extremely Large VLDL	0.02893656	4.37E-18	3.06E-17	27.087474
Glucose	0.02889842	3.62E-06	8.56E-06	0.24208158
HDL Cholesterol	-0.0283004	8.05E-14	4.10E-13	17.4118373
Docosahexaenoic Acid	-0.0264563	1.93E-39	8.10E-38	75.8521734
Glutamine	-0.0263136	1.36E-43	7.64E-42	85.356893
Phospholipids in Large HDL	-0.0258977	5.27E-17	3.28E-16	24.6314221
Cholesteryl Esters in HDL	-0.0256823	6.91E-14	3.63E-13	17.5615845
Total Cholesterol	-0.0242049	1.52E-06	3.82E-06	1.07447119
Total Esterified Cholesterol	-0.0235938	3.73E-07	1.01E-06	2.42961206
Total Lipids in Very Large VLDL	0.02279391	1.43E-11	6.00E-11	12.330189
Total Lipids in HDL	-0.0220189	3.88E-07	1.04E-06	2.39117461
Clinical LDL Cholesterol	-0.0211544	0.001	0.002	-5.1339122
Total Lipids in Very Large HDL	-0.0205858	4.43E-27	5.89E-26	47.5836648
Glycine	-0.0204103	1.02E-32	2.84E-31	60.4716346
Total Lipids in Large VLDL	0.01918751	2.87E-06	7.08E-06	0.46685771
Cholesterol in Large LDL	-0.018919	3.95E-06	9.09E-06	0.1592954
Cholesterol in IDL	-0.0179266	1.69E-07	4.82E-07	3.19346896
Triglycerides in Very Large VLDL	0.01621914	3.98E-13	1.92E-12	15.8410938
Total Lipids in IDL	-0.016215	2.29E-05	4.92E-05	-1.5174587
Total Lipids in Large LDL	-0.016089	3.68E-04	6.58E-04	-4.1414322

LDL Cholesterol	-0.0160019	0.001	0.002	-5.3791059
Cholesteryl Esters in IDL	-0.0157404	5.23E-08	1.51E-07	4.3298298
Phospholipids in VLDL	0.01548044	5.78E-05	1.19E-04	-2.3985012
Total Cholesterol Minus HDL-C	-0.0152557	0.012	0.018	-7.3410927
Linoleic Acid	-0.014789	0.003	0.005	-6.1131243
Degree of Unsaturation	-0.0147813	4.10E-38	1.38E-36	72.8116247
Cholesteryl Esters in Large LDL	-0.014255	5.12E-05	1.08E-04	-2.2833486
Total Free Cholesterol	-0.0142026	7.59E-05	1.54E-04	-2.6566494
Total Lipids in Small HDL	0.01280071	3.09E-08	9.28E-08	4.83935415
Phosphatidylcholines	-0.0126695	5.95E-04	0.001	-4.5888637
Free Cholesterol in LDL	-0.0126052	6.31E-07	1.63E-06	1.92240234
Phospholipids in Very Large HDL	-0.0120921	1.08E-24	1.07E-23	42.1376428
Total Lipids in LDL	-0.0119625	0.026	0.038	-8.0223872
Triglycerides in Large VLDL	0.01152445	3.24E-06	7.78E-06	0.3490816
Phospholipids in HDL	-0.011425	9.04E-04	0.002	-4.977295
Polyunsaturated Fatty Acids	-0.0111398	0.010	0.015	-7.1990365
Cholesteryl Esters in LDL	-0.010968	0.014	0.020	-7.4416501
Free Cholesterol in HDL	-0.0107046	4.05E-12	1.75E-11	13.5644926
Apolipoprotein A1	-0.0106328	1.25E-04	2.38E-04	-3.1244944
Free Cholesterol in Large LDL	-0.010609	4.20E-09	1.31E-08	6.77859037
Phospholipids in Small HDL	0.01038247	3.14E-09	9.94E-09	7.06373784
Cholesterol in Chylomicrons and Extremely Large VLDL	0.01025663	7.32E-17	4.39E-16	24.3067333
Cholesterol in Very Large HDL	-0.0101553	2.04E-28	3.80E-27	50.6392304
Triglycerides in Medium VLDL	0.010141	4.29E-04	7.59E-04	-4.2852256
3-Hydroxybutyrate	0.00956655	2.09E-08	6.38E-08	5.2198655
Free Cholesterol in Large HDL	-0.0094082	7.60E-22	6.38E-21	35.6452748
Cholesteryl Esters in Very Large HDL	-0.0084008	2.75E-29	6.60E-28	52.6249865
Phospholipids in Chylomicrons and Extremely Large VLDL	0.00839595	1.99E-19	1.59E-18	30.1375543
Triglycerides in LDL	0.00816701	7.40E-13	3.46E-12	15.232002
Cholesterol in Medium HDL	-0.0080739	4.79E-04	8.38E-04	-4.3874332
Free Cholesterol in VLDL	0.00804012	0.002	0.003	-5.7721711
Total Concentration of Branched-Chain Amino Acids (Leucine + Isoleucine + Valine)	0.00787455	5.79E-05	1.19E-04	-2.4000932
Total Lipids in Small VLDL	0.00786631	0.008	0.012	-6.9321357
Total Cholines	-0.0076505	0.031	0.043	-8.1548776
Triglycerides in Small VLDL	0.00755425	6.41E-06	1.46E-05	-0.3043787
Alanine	0.00736739	1.14E-04	2.23E-04	-3.0395071
Valine	0.00728273	8.17E-11	3.05E-10	10.6236412

Spectrometer-corrected alanine	0.00704404	4.95E-05	1.05E-04	-2.2510592
Cholesteryl Esters in Medium HDL	-0.0067785	6.41E-04	0.001	-4.6591789
Phospholipids in IDL	-0.006472	2.07E-05	4.52E-05	-1.4240204
Phospholipids in Large VLDL	0.00638641	3.89E-08	1.15E-07	4.61778468
Average Diameter for VLDL Particles	0.00633401	5.06E-11	2.02E-10	11.0921442
Pyruvate	0.00597087	1.02E-10	3.73E-10	10.4061167
Free Cholesterol in IDL	-0.0058989	1.58E-05	3.49E-05	-1.1660727
Cholesterol in Medium VLDL	-0.0057965	8.21E-04	0.001	-4.888237
Average Diameter for HDL Particles	-0.0057194	2.03E-25	2.27E-24	43.7950444
Cholesteryl Esters in Chylomicrons and Extremely Large VLDL	0.00556072	2.37E-15	1.28E-14	20.8802339
Triglycerides in Large LDL	0.00528253	1.64E-12	7.24E-12	14.4532274
Phospholipids in Large LDL	-0.0052499	0.005	0.008	-6.5714986
Cholesteryl Esters in Medium VLDL	-0.0052023	1.19E-06	3.03E-06	1.31075833
Phospholipids in Very Large VLDL	0.00509494	7.61E-11	2.90E-10	10.6930747
Free Cholesterol in Chylomicrons and Extremely Large VLDL	0.00506722	2.81E-18	2.05E-17	27.5241178
Cholesterol in Large VLDL	0.00492543	3.44E-04	6.28E-04	-4.0772794
Tyrosine	0.00491829	8.33E-29	1.75E-27	51.526521
Triglycerides in HDL	0.00488579	2.34E-04	4.34E-04	-3.7164534
Triglycerides in IDL	0.00459227	1.70E-09	5.60E-09	7.65987452
Cholesterol in Very Large VLDL	0.00426785	6.03E-07	1.58E-06	1.96579501
Triglycerides in Small HDL	0.00422994	1.62E-15	9.09E-15	21.2533179
Cholesterol in Small HDL	0.00401483	0.003	0.005	-6.0829092
Triglycerides in Very Small VLDL	0.00400789	5.64E-10	1.93E-09	8.73660781
Free Cholesterol in Large VLDL	0.00386952	2.61E-07	7.18E-07	2.77524998
Cholesteryl Esters in Small HDL	0.00370873	0.001	0.002	-5.2582063
Cholesteryl Esters in Very Small VLDL	-0.0036461	9.17E-05	1.83E-04	-2.8353687
Cholesterol in Very Small VLDL	-0.0033546	0.008	0.011	-6.9258928
Phenylalanine	0.00290359	3.36E-17	2.17E-16	25.0761593
Free Cholesterol in Medium LDL	-0.0028706	0.002	0.003	-5.5031218
Free Cholesterol in Very Large VLDL	0.00263937	2.42E-09	7.82E-09	7.31553795
Triglycerides in Medium HDL	0.00261817	7.66E-06	1.72E-05	-0.4744861
Free Cholesterol in Medium HDL	-0.0024175	1.70E-04	3.21E-04	-3.4156555
Triglycerides in Medium LDL	0.00233758	4.01E-13	1.92E-12	15.8346868
Histidine	-0.0021779	2.39E-10	8.53E-10	9.57599886
Free Cholesterol in Very Large HDL	-0.0020595	4.99E-22	4.41E-21	36.0611445
Cholesteryl Esters in Very Large VLDL	0.00175753	1.03E-04	2.03E-04	-2.9424423
Triglycerides in Large HDL	-0.0014349	1.22E-04	2.35E-04	-3.1012789

Free Cholesterol in Small LDL	-0.0013519	3.48E-04	6.28E-04	-4.0877656
Triglycerides in Small LDL	0.00120708	1.93E-11	7.91E-11	12.0345292
Average Diameter for LDL Particles	-6.36E-04	2.99E-06	7.29E-06	0.42525199
Concentration of Large HDL Particles	-2.24E-04	7.53E-25	7.91E-24	42.4939857
Total Concentration of Lipoprotein Particles	-1.90E-04	0.010	0.015	-7.1680778
Concentration of HDL Particles	-1.73E-04	0.014	0.021	-7.4911876
Concentration of Small HDL Particles	1.54E-04	2.35E-04	4.34E-04	-3.7215664
Concentration of Medium HDL Particles	-7.63E-05	0.004	0.007	-6.4065381
Concentration of Very Large HDL Particles	-3.04E-05	1.74E-27	2.66E-26	48.5096435
Concentration of Large LDL Particles	-1.59E-05	0.004	0.007	-6.441145
Concentration of VLDL Particles	3.32E-06	0.027	0.038	-8.0255874

Positive log fold-change (log2FC) indicates higher levels in the Confirmed Sarcopenia group. FDR adjusted *P* values calculated using Benjamini–Hochberg correction. Linear modelling was adjusted for age, sex, ethnicity, education, deprivation index, protein intake, physical activity, smoking, alcohol use, diabetes, hypertension, coronary heart disease, and chronic kidney disease.

Table S8. Significant differential metabolites (FDR < 0.05) between Low HGS/BMI only (N=18,796) and Both Normal group(N=243,858).

**Metabolite**	**Log2FC**	***P* Value**	**adj. *P* Value**	**B**
Triglycerides in VLDL	0.07908572	1.93E-208	1.29E-207	463.072952
Total Triglycerides	0.07881679	6.91E-208	4.30E-207	461.797719
Total Lipids in VLDL	0.07875507	1.06E-156	4.14E-156	344.078803
Monounsaturated Fatty Acids	0.07007309	9.63E-210	6.74E-209	466.066209
Total Lipids in Large HDL	-0.0595274	1.77E-252	2.98E-251	564.374621
Total Lipids in Chylomicrons and Extremely Large VLDL	0.05674008	7.94E-264	2.67E-262	590.483783
Saturated Fatty Acids	0.04404248	3.25E-103	9.42E-103	221.132042
Cholesterol in Large HDL	-0.0437681	8.64E-300	4.84E-298	673.228804
HDL Cholesterol	-0.0423218	1.99E-234	1.96E-233	522.851474
Cholesteryl Esters in HDL	-0.0395738	5.54E-250	8.47E-249	558.633567
Triglycerides in Chylomicrons and Extremely Large VLDL	0.03945699	5.09E-257	9.51E-256	574.822598
Total Lipids in Large VLDL	0.03903641	2.31E-169	1.05E-168	373.197014
Total Lipids in Very Large VLDL	0.03727128	1.96E-226	1.74E-225	504.458578
Cholesteryl Esters in Large HDL	-0.0370848	1.27E-312	1.07E-310	702.756473
Total Fatty Acids	0.0365562	1.93E-70	4.50E-70	145.863622
Total Lipids in HDL	-0.0351162	6.91E-124	2.07E-123	268.640673
Phospholipids in Large HDL	-0.0324305	2.28E-208	1.48E-207	462.902927
Albumin	-0.0321044	5.36E-196	3.00E-195	434.449091
Phospholipids in VLDL	0.03207743	2.45E-130	8.24E-130	283.466743
Glycoprotein Acetyls	0.02970274	0	0	875.755916
Triglycerides in Very Large VLDL	0.02525356	5.23E-236	5.49E-235	526.484979
VLDL Cholesterol	0.02469391	1.17E-57	2.52E-57	116.53307
Triglycerides in Medium VLDL	0.02372999	3.03E-127	9.79E-127	276.359067
Triglycerides in Large VLDL	0.02307134	1.11E-161	4.64E-161	355.534758
Total Lipids in Medium VLDL	0.02258712	9.93E-57	2.11E-56	114.402145
Phospholipids in HDL	-0.0217944	1.46E-76	3.51E-76	159.911994
Total Esterified Cholesterol	-0.0214303	3.70E-41	7.22E-41	78.713164
Total Lipids in Small VLDL	0.02029296	1.58E-89	4.08E-89	189.691122
Total Lipids in Very Large HDL	-0.020142	1.12E-210	8.18E-210	468.215558
Total Cholesterol	-0.0197984	2.47E-30	4.20E-30	53.9445084
Apolipoprotein A1	-0.0193604	1.14E-92	3.09E-92	196.907772
Free Cholesterol in VLDL	0.01887139	5.17E-98	1.47E-97	209.18132
Glucose	0.01881494	3.61E-18	5.52E-18	26.2058864
Cholesterol in Medium HDL	-0.0187427	4.73E-124	1.45E-123	269.018551
Docosahexaenoic Acid	-0.0181767	2.35E-153	8.98E-153	336.386016

Glucose-lactate	0.0181538	7.13E-27	1.19E-26	46.0423925
Cholesteryl Esters in Medium HDL	-0.0164399	2.92E-129	9.62E-129	280.992926
Total Lipids in Medium HDL	-0.0158234	5.35E-49	1.08E-48	96.6751373
Clinical LDL Cholesterol	-0.0155926	2.28E-12	3.14E-12	13.0568326
Triglycerides in Small VLDL	0.01507449	2.15E-151	8.01E-151	331.878864
Omega-3 Fatty Acids	-0.0148932	7.27E-24	1.20E-23	39.1768878
Degree of Unsaturation	-0.0144341	2.20E-295	9.24E-294	663.090559
Cholesterol in IDL	-0.0143642	2.91E-34	5.42E-34	62.9311593
Free Cholesterol in HDL	-0.014122	1.42E-158	5.67E-158	348.388276
Cholesterol in Chylomicrons and Extremely Large VLDL	0.01397858	3.75E-239	4.20E-238	533.71766
Cholesterol in Large LDL	-0.0136952	2.24E-22	3.66E-22	35.7821823
Cholesteryl Esters in VLDL	0.01253843	1.37E-31	2.37E-31	56.8170361
Glutamine	-0.0123306	7.39E-80	1.85E-79	167.48221
Cholesteryl Esters in IDL	-0.0119848	1.44E-33	2.59E-33	61.3442675
Phospholipids in Very Large HDL	-0.0119528	2.78E-195	1.51E-194	432.803884
Phospholipids in Large VLDL	0.01183339	3.96E-193	2.02E-192	427.852606
Glycine	-0.0117913	4.36E-90	1.16E-89	190.975271
Cholesterol in Large VLDL	0.01161264	7.63E-134	2.62E-133	291.528575
Total Concentration of Branched-Chain Amino Acids (Leucine + Isoleucine + Valine)	0.01139469	1.64E-64	3.73E-64	132.252264
Phospholipids in Chylomicrons and Extremely Large VLDL	0.01101527	1.62E-257	3.41E-256	575.964332
Free Cholesterol in LDL	-0.0109515	1.87E-36	3.53E-36	67.9444518
Free Cholesterol in Large HDL	-0.010837	5.74E-232	5.36E-231	517.189797
Total Lipids in IDL	-0.0106779	4.52E-16	6.66E-16	21.4410924
Triglycerides in LDL	0.01060513	7.04E-161	2.88E-160	353.686676
Triglycerides in HDL	0.01052686	1.14E-117	3.35E-117	254.353866
Total Lipids in Large LDL	-0.0104701	1.45E-11	1.93E-11	11.2459865
Cholesterol in Very Large HDL	-0.0103972	2.05E-242	2.65E-241	541.222997
Lactate	0.01022835	4.74E-05	5.85E-05	-3.280853
Average Diameter for VLDL Particles	0.01006346	1.30E-203	7.82E-203	451.963268
Phosphatidylcholines	-0.0092981	2.05E-13	2.86E-13	15.4239644
LDL Cholesterol	-0.0092055	8.59E-08	1.09E-07	2.77811691
Cholesteryl Esters in Large LDL	-0.0091678	3.14E-14	4.51E-14	17.265469
Free Cholesterol in Large LDL	-0.0090864	1.02E-48	2.03E-48	96.0358083
Total Cholines	-0.0090046	1.28E-13	1.81E-13	15.8857454
Cholesteryl Esters in Very Large HDL	-0.00882	3.46E-263	9.69E-262	589.013269
Phospholipids in Very Large VLDL	0.00847046	4.19E-217	3.20E-216	482.999117
Total Lipids in Very Small VLDL	0.00841786	9.59E-34	1.75E-33	61.7447259

Cholesterol in Very Large VLDL	0.00802406	1.41E-164	6.09E-164	362.187648
Cholesteryl Esters in Chylomicrons and Extremely Large VLDL	0.00774849	8.80E-226	7.39E-225	502.960577
Valine	0.00756302	4.77E-86	1.21E-85	181.697631
Total Free Cholesterol	-0.0073932	1.97E-09	2.57E-09	6.44371341
Free Cholesterol in Large VLDL	0.00727533	5.89E-175	2.83E-174	386.05906
Triglycerides in IDL	0.00673766	1.94E-145	6.79E-145	318.183735
Free Cholesterol in Chylomicrons and Extremely Large VLDL	0.00669617	7.14E-245	1.00E-243	546.877595
Sphingomyelins	-0.0066788	1.24E-39	2.37E-39	75.2183374
Triglycerides in Large LDL	0.00664526	1.01E-146	3.60E-146	321.138124
Total Lipids in Small HDL	0.00658589	9.88E-17	1.47E-16	22.9401326
Remnant Cholesterol (Non-HDL, Non-LDL -Cholesterol)	0.0063746	2.10E-04	2.50E-04	-4.684844
Phospholipids in Medium HDL	-0.0062127	1.20E-20	1.88E-20	31.8445612
Triglycerides in Very Small VLDL	0.00619054	2.38E-170	1.11E-169	375.466657
Total Lipids in Lipoprotein Particles	0.00617316	5.65E-04	6.64E-04	-5.6130977
Triglycerides in Small HDL	0.00605499	1.95E-241	2.33E-240	538.974989
Average Diameter for HDL Particles	-0.005938	3.19E-221	2.55E-220	492.471914
Cholesterol in Small VLDL	0.00589425	3.65E-34	6.75E-34	62.7033387
Linoleic Acid	-0.0057786	7.58E-04	8.84E-04	-5.8856197
Phospholipids in Small HDL	0.00573246	1.51E-21	2.42E-21	33.8949264
Free Cholesterol in IDL	-0.005484	1.32E-31	2.30E-31	56.8571844
Phospholipids in Medium VLDL	0.00534954	3.14E-31	5.38E-31	55.9934083
Total Lipids in Medium LDL	0.00530766	1.68E-06	2.12E-06	-0.0923642
Cholesteryl Esters in Medium LDL	0.00510773	2.67E-12	3.65E-12	12.9007649
Cholesteryl Esters in Small VLDL	0.00491723	1.27E-50	2.63E-50	100.401356
Cholesteryl Esters in Large VLDL	0.00490194	1.37E-89	3.60E-89	189.832686
Triglycerides in Medium HDL	0.0048182	7.26E-127	2.30E-126	275.487499
Total Lipids in LDL	-0.0046496	0.012	0.013	-8.3995361
Free Cholesterol in Very Large VLDL	0.00457848	4.78E-199	2.77E-198	441.465346
Apolipoprotein B	0.0045343	8.57E-05	1.04E-04	-3.8425482
Free Cholesterol in Medium HDL	-0.0045131	8.84E-94	2.44E-93	199.457426
Polyunsaturated Fatty Acids	-0.0044026	0.003	0.004	-7.1908856
Total Phospholipids in Lipoprotein Particles	-0.0043844	3.49E-04	4.13E-04	-5.16204
Phospholipids in Small VLDL	0.00399928	3.39E-40	6.54E-40	76.5103409
Phospholipids in Very Small VLDL	0.00399349	3.82E-53	8.02E-53	106.179981
Phosphoglycerides	-0.0038373	0.003	0.003	-7.1195927
Phospholipids in IDL	-0.0038284	2.12E-13	2.95E-13	15.3866164
Cholesteryl Esters in Very Large VLDL	0.0036752	3.50E-124	1.09E-123	269.319414

Phospholipids in Large LDL	-0.0036415	1.52E-08	1.97E-08	4.45698126
Cholesteryl Esters in LDL	-0.0036376	0.017	0.019	-8.7136009
Leucine	0.00329049	1.01E-32	1.81E-32	59.4037757
Tyrosine	0.00312742	2.14E-94	5.98E-94	200.874467
Cholesteryl Esters in Medium VLDL	-0.0031029	3.03E-17	4.54E-17	24.1074284
Triglycerides in Medium LDL	0.00304615	2.38E-165	1.05E-164	363.968182
Alanine	0.0027903	1.97E-05	2.45E-05	-2.4472651
Cholesterol in Medium LDL	0.00270364	0.003	0.003	-6.988405
Isoleucine	0.00263831	3.04E-45	6.01E-45	88.070568
Total Lipids in Small LDL	0.00255921	3.51E-06	4.39E-06	-0.797318
Pyruvate	0.00247167	4.45E-15	6.50E-15	19.1889038
Free Cholesterol in Medium LDL	-0.0024386	5.22E-15	7.55E-15	19.0323724
Spectrometer-corrected alanine	0.00226349	1.42E-04	1.71E-04	-4.3168082
Phenylalanine	0.00223641	1.18E-78	2.93E-78	164.715721
Free Cholesterol in Medium VLDL	0.00222313	6.70E-14	9.54E-14	16.521128
Cholesteryl Esters in Small LDL	0.00213835	1.84E-11	2.44E-11	11.0097711
Citrate	-0.0021346	2.62E-58	5.72E-58	118.022068
Free Cholesterol in Very Large HDL	-0.0019056	2.83E-151	1.03E-150	331.604185
Phospholipids in LDL	-0.0018643	0.046	0.050	-9.5740575
Triglycerides in Small LDL	0.00179676	3.21E-184	1.59E-183	407.362671
Phospholipids in Medium LDL	0.00148739	1.70E-04	2.04E-04	-4.485876
Free Cholesterol in Very Small VLDL	0.00143475	1.33E-21	2.15E-21	34.0180517
Free Cholesterol in Small VLDL	0.00134866	3.49E-12	4.72E-12	12.6406669
Cholesteryl Esters in Small HDL	-0.0012575	0.001	0.002	-6.4356593
Free Cholesterol in Small LDL	-0.0012107	1.03E-20	1.63E-20	31.9991543
Cholesterol in Small HDL	-0.0011505	0.013	0.014	-8.4772839
Histidine	-0.0010261	2.81E-18	4.37E-18	26.4529863
Cholesterol in Small LDL	9.58E-04	0.018	0.020	-8.7774045
Cholesteryl Esters in Very Small VLDL	-9.49E-04	0.003	0.003	-7.1513852
Average Diameter for LDL Particles	-7.72E-04	1.43E-62	3.21E-62	127.801333
Creatinine	4.44E-04	0.004	0.005	-7.4343344
Concentration of HDL Particles	-4.26E-04	2.57E-69	5.91E-69	143.283344
Total Concentration of Lipoprotein Particles	-4.08E-04	6.17E-59	1.36E-58	119.463903
Triglycerides in Very Large HDL	3.54E-04	2.37E-32	4.20E-32	58.5576244
Triglycerides in Large HDL	3.50E-04	0.006	0.007	-7.8241532
Concentration of Large HDL Particles	-2.54E-04	1.32E-258	3.17E-257	578.471576
Concentration of Medium HDL Particles	-1.70E-04	4.44E-77	1.08E-76	161.102494

Concentration of Very Large HDL Particles	-2.84E-05	3.32E-195	1.75E-194	432.626848
Concentration of VLDL Particles	9.45E-06	8.12E-76	1.92E-75	158.203664
Concentration of LDL Particles	7.21E-06	0.019	0.021	-8.8087091
Concentration of Medium LDL Particles	5.30E-06	3.09E-10	4.05E-10	8.252951
Concentration of Small VLDL Particles	2.95E-06	2.68E-49	5.50E-49	97.361963
Concentration of Small LDL Particles	2.95E-06	8.39E-12	1.13E-11	11.7798178
Concentration of Very Small VLDL Particles	1.75E-06	7.83E-18	1.18E-17	25.4420155
Concentration of Large VLDL Particles	1.73E-06	4.41E-30	7.42E-30	53.370009
Concentration of Medium VLDL Particles	1.69E-06	3.61E-18	5.52E-18	26.2051985
Concentration of Very Large VLDL Particles	7.84E-07	2.53E-08	3.24E-08	3.96466791
Concentration of Chylomicrons and Extremely Large VLDL Particles	5.51E-07	7.65E-05	9.38E-05	-3.7351891

Positive log fold-change (log2FC) indicates higher levels in the Low HGS/BMI only group. FDR adjusted *P* values calculated using Benjamini–Hochberg correction. Linear modelling was adjusted for age, sex, ethnicity, education, deprivation index, protein intake, physical activity, smoking, alcohol use, diabetes, hypertension, coronary heart disease, and chronic kidney disease.

Table S9. Detailed results of pathways analysis of differential metabolites between Confirmed Sarcopenia vs Reference

	Total	Expected	Hits	Raw *P*	-LOG10(*P*)	Holm adjust	FDR	Impact
**Phenylalanine, tyrosine and tryptophan biosynthesis**	**4**	0.030	2	0.000	3.51	0.025	**0.025**	**1.00**
Alanine, aspartate and glutamate metabolism	28	0.211	3	0.001	3.02	0.076	0.029	0.11
**Phenylalanine metabolism**	**8**	0.060	2	0.001	2.85	0.111	**0.029**	**0.36**
Glyoxylate and dicarboxylate metabolism	32	0.241	3	0.001	2.84	0.111	0.029	0.08
Pyruvate metabolism	23	0.173	2	0.012	1.92	0.917	0.175	0.19
Neomycin, kanamycin and gentamicin biosynthesis	2	0.015	1	0.015	1.82	1.000	0.175	0.00
Glycolysis or Gluconeogenesis	26	0.196	2	0.015	1.81	1.000	0.175	0.10
Lipoic acid metabolism	28	0.211	2	0.018	1.75	1.000	0.177	0.00
Glycine, serine and threonine metabolism	33	0.249	2	0.024	1.62	1.000	0.215	0.26
Biosynthesis of unsaturated fatty acids	36	0.271	2	0.028	1.55	1.000	0.228	0.00
Linoleic acid metabolism	5	0.038	1	0.037	1.43	1.000	0.253	1.00
Tyrosine metabolism	42	0.317	2	0.038	1.42	1.000	0.253	0.14
Nitrogen metabolism	6	0.045	1	0.044	1.35	1.000	0.274	0.00
Valine, leucine and isoleucine biosynthesis	8	0.060	1	0.059	1.23	1.000	0.336	0.00
Arginine biosynthesis	14	0.106	1	0.101	1.00	1.000	0.537	0.00
Histidine metabolism	16	0.121	1	0.115	0.94	1.000	0.537	0.22
Starch and sucrose metabolism	18	0.136	1	0.128	0.89	1.000	0.537	0.42
Ubiquinone and other terpenoid-quinone biosynthesis	19	0.143	1	0.135	0.87	1.000	0.537	0.00
Selenocompound metabolism	20	0.151	1	0.141	0.85	1.000	0.537	0.00
Pantothenate and CoA biosynthesis	20	0.151	1	0.141	0.85	1.000	0.537	0.00
Citrate cycle (TCA cycle)	20	0.151	1	0.141	0.85	1.000	0.537	0.05
beta-Alanine metabolism	21	0.158	1	0.148	0.83	1.000	0.537	0.00
One carbon pool by folate	26	0.196	1	0.180	0.75	1.000	0.616	0.04
Galactose metabolism	27	0.204	1	0.186	0.73	1.000	0.616	0.03
Glutathione metabolism	28	0.211	1	0.192	0.72	1.000	0.616	0.09
Porphyrin metabolism	31	0.234	1	0.211	0.68	1.000	0.649	0.00
Cysteine and methionine metabolism	33	0.249	1	0.223	0.65	1.000	0.661	0.00
Arginine and proline metabolism	36	0.271	1	0.241	0.62	1.000	0.688	0.00
Pyrimidine metabolism	39	0.294	1	0.258	0.59	1.000	0.704	0.00
Valine, leucine and isoleucine degradation	40	0.302	1	0.264	0.58	1.000	0.704	0.00
Primary bile acid biosynthesis	46	0.347	1	0.297	0.53	1.000	0.768	0.01
Purine metabolism	70	0.528	1	0.418	0.38	1.000	1.000	0.00

Table S10. Detailed results of pathways analysis of differential metabolites between Low HGS only vs Reference

	Total	Expected	Hits	Raw *P*	-LOG10(*P*)	Holm adjust	FDR	Impact
Valine, leucine and isoleucine biosynthesis	8	0.080	3	4.53E-05	4.34	0.004	0.004	0.00
Alanine, aspartate and glutamate metabolism	28	0.281	4	0.000	3.92	0.010	0.005	0.11
Glyoxylate and dicarboxylate metabolism	32	0.322	4	0.000	3.68	0.016	0.006	0.16
**Phenylalanine, tyrosine and tryptophan biosynthesis**	**4**	0.040	2	0.001	3.25	0.043	**0.011**	**1.00**
**Linoleic acid metabolism**	**5**	0.050	2	0.001	3.03	0.071	**0.015**	**1.00**
**Phenylalanine metabolism**	**8**	0.080	2	0.003	2.59	0.192	**0.034**	**0.36**
Valine, leucine and isoleucine degradation	40	0.402	3	0.007	2.18	0.486	0.075	0.00
Citrate cycle (TCA cycle)	20	0.201	2	0.016	1.79	1	0.162	0.14
Neomycin, kanamycin and gentamicin biosynthesis	2	0.020	1	0.020	1.70	1	0.170	0.00
Pyruvate metabolism	23	0.231	2	0.021	1.67	1	0.170	0.19
Glycolysis or Gluconeogenesis	26	0.261	2	0.027	1.57	1	0.195	0.10
Lipoic acid metabolism	28	0.281	2	0.031	1.51	1	0.205	0.00
Glycine, serine and threonine metabolism	33	0.332	2	0.042	1.38	1	0.257	0.26
Biosynthesis of unsaturated fatty acids	36	0.362	2	0.049	1.31	1	0.280	0.00
Nitrogen metabolism	6	0.060	1	0.059	1.23	1	0.314	0.00
Tyrosine metabolism	42	0.422	2	0.065	1.19	1	0.323	0.14
alpha-Linolenic acid metabolism	13	0.131	1	0.124	0.91	1	0.581	0.00
Arginine biosynthesis	14	0.141	1	0.132	0.88	1	0.588	0.00
Histidine metabolism	16	0.161	1	0.150	0.82	1	0.631	0.22
Starch and sucrose metabolism	18	0.181	1	0.167	0.78	1	0.640	0.42
Ubiquinone and other terpenoid-quinone biosynthesis	19	0.191	1	0.176	0.76	1	0.640	0.00
Selenocompound metabolism	20	0.201	1	0.184	0.74	1	0.640	0.00
Pantothenate and CoA biosynthesis	20	0.201	1	0.184	0.74	1	0.640	0.00
beta-Alanine metabolism	21	0.211	1	0.192	0.72	1	0.641	0.00
One carbon pool by folate	26	0.261	1	0.233	0.63	1	0.735	0.04
Galactose metabolism	27	0.271	1	0.240	0.62	1	0.735	0.03
Glutathione metabolism	28	0.281	1	0.248	0.61	1	0.735	0.09
Porphyrin metabolism	31	0.312	1	0.271	0.57	1	0.774	0.00
Cysteine and methionine metabolism	33	0.332	1	0.286	0.54	1	0.789	0.00
Arginine and proline metabolism	36	0.362	1	0.308	0.51	1	0.794	0.00
Glycerophospholipid metabolism	36	0.362	1	0.308	0.51	1	0.794	0.09
Pyrimidine metabolism	39	0.392	1	0.329	0.48	1	0.822	0.00
Arachidonic acid metabolism	44	0.442	1	0.363	0.44	1	0.879	0.00
Primary bile acid biosynthesis	46	0.462	1	0.376	0.42	1	0.884	0.01
Purine metabolism	70	0.704	1	0.515	0.29	1	1	0.00

Table S11. Significant differential metabolites (FDR adjusted *P* value < 0.05) between Confirmed Sarcopenia(N=209) and Both Normal (N= 12,131) (reference) at instance 1.

Metabolite	Log2FC	*P* Value	adj. *P* Value	B
Monounsaturated Fatty Acids	0.081706912	2.19E-05	0.0001	-0.337752941
Clinical LDL Cholesterol	-0.078571602	1.44E-04	5.75E-04	-2.121346243
Triglycerides in VLDL	0.076654538	7.99E-04	0.002	-3.726526599
Total Lipids in Large HDL	-0.076126292	5.61E-06	5.24E-05	0.960189399
Total Triglycerides	0.075431935	8.16E-04	0.002	-3.745797651
Total Cholesterol	-0.06757015	3.64E-05	1.97E-04	-0.822944385
Total Lipids in Chylomicrons and Extremely Large VLDL	0.066641408	2.30E-06	2.49E-05	1.817069496
Glucose-lactate	0.066483915	6.99E-07	1.07E-05	2.962012499
Total Esterified Cholesterol	-0.063721564	2.50E-05	1.41E-04	-0.464467754
Lactate	0.062502713	0.005	0.010	-5.321536694
Total Cholesterol Minus HDL-C	-0.061287097	0.002	0.004	-4.395990883
Glucose	0.061123484	7.23E-06	6.08E-05	0.717749702
Cholesterol in Large HDL	-0.057939446	4.41E-07	9.27E-06	3.404593465
LDL Cholesterol	-0.052263333	0.001	0.003	-3.966243889
Total Lipids in LDL	-0.050353369	0.003	0.008	-5.00692018
Total Lipids in Large LDL	-0.049496694	6.03E-04	0.002	-3.463743954
Linoleic Acid	-0.04922802	0.001	0.004	-4.294854932
Total Lipids in IDL	-0.049213231	7.77E-05	3.60E-04	-1.540350041
Cholesterol in IDL	-0.049116536	1.11E-05	7.76E-05	0.309592844
Cholesterol in Large LDL	-0.049053652	1.86E-04	6.96E-04	-2.365721996
Cholesteryl Esters in Large HDL	-0.04864658	3.30E-07	7.92E-06	3.685591551
Triglycerides in Chylomicrons and Extremely Large VLDL	0.048581021	1.04E-06	1.46E-05	2.576049566
HDL Cholesterol	-0.046980654	1.30E-04	5.34E-04	-2.02964798
Glycoprotein Acetyls	0.046023177	1.76E-13	1.48E-11	17.77910892
Albumin	-0.043263726	6.11E-07	1.03E-05	3.090953046
Remnant Cholesterol (Non-HDL, Non-LDL -Cholesterol)	-0.042494753	0.008	0.016	-5.825647887
Cholesteryl Esters in HDL	-0.042417502	1.28E-04	5.34E-04	-2.012683212
Saturated Fatty Acids	0.041780238	0.016	0.028	-6.423711361
Total Free Cholesterol	-0.040952916	4.16E-04	0.001	-3.118560185
Cholesteryl Esters in IDL	-0.040897706	1.37E-05	8.89E-05	0.104266021
Cholesteryl Esters in LDL	-0.039206802	0.005	0.012	-5.481296721
Phospholipids in Large HDL	-0.037866509	1.70E-04	6.48E-04	-2.278237032
Cholesteryl Esters in Large LDL	-0.037271488	9.11E-04	0.003	-3.84741124
Total Lipids in Very Large VLDL	0.036292093	4.27E-04	0.001	-3.142798879

Total Lipids in HDL	-0.035198509	0.011	0.020	-6.095382431
Free Cholesterol in LDL	-0.033302706	3.80E-05	1.99E-04	-0.862884041
Total Lipids in Very Large HDL	-0.033210529	2.77E-07	7.76E-06	3.853438214
Total Lipids in Large VLDL	0.032229495	0.012	0.021	-6.159256374
Triglycerides in Very Large VLDL	0.027721149	4.93E-05	2.36E-04	-1.109592777
Apolipoprotein B	-0.026150401	0.014	0.025	-6.320199924
Free Cholesterol in Large LDL	-0.025269098	1.38E-05	8.89E-05	0.103429137
Docosahexaenoic Acid	-0.024392286	1.24E-04	5.34E-04	-1.982639587
Phospholipids in LDL	-0.023703503	0.006	0.013	-5.58133823
Triglycerides in Large VLDL	0.022048864	0.005	0.010	-5.314608427
Glutamine	-0.022013556	4.56E-05	2.25E-04	-1.035824141
Glycine	-0.019832759	1.56E-04	6.09E-04	-2.197900662
Phospholipids in Large LDL	-0.019027508	0.001	0.004	-4.303712818
Phospholipids in Very Large HDL	-0.019012346	1.68E-06	2.17E-05	2.119380454
Cholesterol in Medium VLDL	-0.018282465	9.53E-04	0.003	-3.889230194
Degree of Unsaturation	-0.018135267	6.86E-08	2.30E-06	5.205183547
Phospholipids in IDL	-0.017742533	3.22E-04	0.001	-2.879994465
Free Cholesterol in IDL	-0.017574941	8.14E-05	3.60E-04	-1.584533505
Free Cholesterol in HDL	-0.017405416	6.68E-04	0.002	-3.559451207
Cholesterol in Very Large HDL	-0.01708739	5.32E-08	2.23E-06	5.451596644
Total Lipids in Small HDL	0.016991643	0.019	0.033	-6.588176865
Pyruvate	0.016571192	7.45E-14	1.25E-11	18.62573145
Phospholipids in Small HDL	0.014871129	0.006	0.013	-5.642320884
Cholesteryl Esters in Medium VLDL	-0.014663431	2.03E-05	1.27E-04	-0.269434772
Spectrometer-corrected alanine	0.01458627	0.006	0.012	-5.511190328
Free Cholesterol in Large HDL	-0.014520497	9.74E-06	7.44E-05	0.433056565
Alanine	0.014176384	0.017	0.029	-6.479680966
Cholesterol in Chylomicrons and Extremely Large VLDL	0.014116929	8.11E-05	3.60E-04	-1.581711096
Cholesteryl Esters in Very Large HDL	-0.013947113	4.74E-08	2.23E-06	5.562220248
Sphingomyelins	-0.013856649	0.004	0.009	-5.179200978
Triglycerides in Small VLDL	0.012895375	0.010	0.020	-6.072325676
Phospholipids in Chylomicrons and Extremely Large VLDL	0.012805063	2.36E-06	2.49E-05	1.792359327
Triglycerides in HDL	0.011402108	0.003	0.008	-5.02377622
Average Diameter for VLDL Particles	0.011241775	1.98E-04	7.23E-04	-2.421996247
Cholesterol in Very Small VLDL	-0.010679313	0.008	0.016	-5.824608493
Phospholipids in Large VLDL	0.010115366	0.005	0.010	-5.334752704
Cholesteryl Esters in Very Small VLDL	-0.009414391	0.002	0.004	-4.45699167

Free Cholesterol in Medium LDL	-0.009234398	0.001	0.003	-4.172805763
Triglycerides in LDL	0.009088189	0.005	0.011	-5.391180448
Valine	0.008577004	0.013	0.023	-6.23103706
Average Diameter for HDL Particles	-0.008279702	2.37E-06	2.49E-05	1.787852883
Cholesterol in Small LDL	-0.008181573	0.027	0.046	-6.914973804
Phospholipids in Very Large VLDL	0.00785684	9.12E-04	0.003	-3.848790732
Free Cholesterol in Chylomicrons and Extremely Large VLDL	0.007536405	8.49E-06	6.79E-05	0.564798752
Triglycerides in Small HDL	0.007133169	7.19E-06	6.08E-05	0.722932704
Cholesteryl Esters in Chylomicrons and Extremely Large VLDL	0.007023682	5.78E-04	0.002	-3.424320193
Acetate	-0.006015683	9.75E-04	0.003	-3.910104004
Triglycerides in Medium HDL	0.005987972	5.22E-04	0.002	-3.330255774
Free Cholesterol in Large VLDL	0.005843761	0.011	0.021	-6.145987136
Triglycerides in Very Small VLDL	0.005664313	0.002	0.006	-4.684759541
Triglycerides in IDL	0.005540663	0.011	0.020	-6.098614656
Triglycerides in Large LDL	0.005506858	0.010	0.019	-5.997587283
Tyrosine	0.005422902	4.15E-05	2.11E-04	-0.945834936
Phenylalanine	0.004596664	2.51E-05	1.41E-04	-0.470237361
Free Cholesterol in Small LDL	-0.004214592	3.79E-04	0.001	-3.030788461
Free Cholesterol in Very Large VLDL	0.003934974	0.003	0.008	-5.027931283
Phospholipids in Small LDL	-0.003763339	0.027	0.046	-6.906120979
Free Cholesterol in Very Large HDL	-0.003666968	5.45E-07	1.02E-05	3.201740328
Citrate	-0.003286836	0.008	0.016	-5.811468102
Triglycerides in Medium LDL	0.002680733	0.003	0.007	-4.981029439
Triglycerides in Small LDL	0.001677433	0.001	0.003	-4.008506198
Average Diameter for LDL Particles	-0.001182228	0.001	0.002	-3.433290687
Concentration of Large HDL Particles	-3.34E-04	4.91E-06	4.85E-05	1.08919205
Concentration of LDL Particles	-6.69E-05	0.020	0.035	-6.651076441
Concentration of Very Large HDL Particles	-4.92E-05	1.06E-05	7.72E-05	0.355513164
Concentration of Large LDL Particles	-4.83E-05	0.009	0.018	-5.954522233
Concentration of IDL Particles	-2.03E-05	0.030	0.049	-6.984406655

Positive log fold-change (log2FC) indicates higher levels in the Confirmed Sarcopenia group. FDR adjusted *P* values calculated using Benjamini–Hochberg correction. Linear modelling was adjusted for age, sex, ethnicity, education, deprivation index, protein intake, physical activity, smoking, alcohol use, diabetes, hypertension, coronary heart disease, and chronic kidney disease.

Abbreviations: FDR, false discovery rate; FC, fold change; HDL, high-density lipoprotein; IDL, intermediate-density lipoprotein; LDL, low-density lipoprotein; VLDL, very-low-density lipoprotein.

Table S12. Significant differential metabolites (FDR < 0.05) at follow-up (Instance 1) according to baseline HGS/BMI status (Low HGS/BMI only: N= 659; Both Normal group: N= 14,514).

**Metabolite**	**Log2FC**	***P* Value**	**adj. *P* Value**	**B**
Total Lipids in Large HDL	-0.0550021	8.77E-09	2.46E-07	6.64098703
Triglycerides in VLDL	0.0517833	9.13E-05	3.41E-04	-2.2533645
Total Triglycerides	0.04814629	2.19E-04	7.15E-04	-3.0766331
Total Lipids in VLDL	0.04800426	0.002	0.004	-4.9025605
HDL Cholesterol	-0.0435255	6.34E-10	5.32E-08	9.20071015
Total Lipids in HDL	-0.0422232	9.74E-08	1.49E-06	4.30564083
Total Lipids in Chylomicrons and Extremely Large VLDL	0.04050946	8.40E-07	6.72E-06	2.22400265
Monounsaturated Fatty Acids	0.03997764	3.20E-04	9.95E-04	-3.4322284
Cholesteryl Esters in HDL	-0.0396672	4.36E-10	5.32E-08	9.56600928
Cholesterol in Large HDL	-0.0381858	5.00E-09	1.68E-07	7.18702667
Cholesteryl Esters in Large HDL	-0.0320867	3.32E-09	1.39E-07	7.58760746
Total Esterified Cholesterol	-0.0318687	2.05E-04	6.89E-04	-3.0153843
Total Cholesterol	-0.0316696	6.50E-04	0.002	-4.0950585
Phospholipids in Large HDL	-0.0309497	6.94E-08	1.17E-06	4.63380907
Albumin	-0.0303164	1.75E-09	9.79E-08	8.21078286
Triglycerides in Chylomicrons and Extremely Large VLDL	0.02878337	7.81E-07	6.56E-06	2.29512139
Phospholipids in HDL	-0.0286314	4.37E-06	2.62E-05	0.64043592
Glucose	0.0286044	3.45E-04	0.001	-3.5042296
Clinical LDL Cholesterol	-0.0269427	0.022	0.038	-7.2739963
Total Lipids in Large VLDL	0.02627349	3.70E-04	0.001	-3.5682994
Apolipoprotein A1	-0.0257832	3.45E-07	3.41E-06	3.08191499
Total Lipids in Very Large VLDL	0.02556952	1.89E-05	8.84E-05	-0.7613633
Total Lipids in Medium HDL	-0.0238258	2.28E-05	1.03E-04	-0.9363499
Glucose-lactate	0.02242116	0.004	0.009	-5.8489763
Cholesterol in Medium HDL	-0.0220813	1.60E-07	1.81E-06	3.82219704
Cholesterol in Large LDL	-0.0209923	0.005	0.010	-5.927213
Omega-3 Fatty Acids	-0.0207275	0.007	0.015	-6.3163827
Total Lipids in Large LDL	-0.0204324	0.013	0.023	-6.7907941
Phosphatidylcholines	-0.0201903	0.002	0.004	-5.0808438
Total Cholines	-0.0201385	0.001	0.003	-4.7135232
Cholesterol in IDL	-0.0201196	0.001	0.004	-4.8192508
Phospholipids in VLDL	0.01973679	0.003	0.007	-5.6153282
Cholesteryl Esters in Medium HDL	-0.0189483	1.62E-07	1.81E-06	3.81531299
Total Lipids in IDL	-0.018779	0.008	0.015	-6.3383806

Glycoprotein Acetyls	0.01843316	3.10E-07	3.25E-06	3.18676691
Docosahexaenoic Acid	-0.0175211	1.46E-06	1.06E-05	1.69536984
Triglycerides in Very Large VLDL	0.01731662	1.33E-05	6.79E-05	-0.4264812
Cholesteryl Esters in IDL	-0.0168277	0.002	0.004	-4.8747049
Phosphoglycerides	-0.0168033	0.010	0.020	-6.6163926
Total Phospholipids in Lipoprotein Particles	-0.0166588	0.010	0.020	-6.6114267
Total Lipids in Very Large HDL	-0.0164213	7.08E-06	3.80E-05	0.17759913
Cholesteryl Esters in Large LDL	-0.0160292	0.012	0.022	-6.7406992
Free Cholesterol in HDL	-0.0159914	4.01E-08	7.48E-07	5.16550933
Total Free Cholesterol	-0.0158511	0.016	0.028	-6.9932024
Triglycerides in Large VLDL	0.01537303	6.48E-04	0.002	-4.0911376
Triglycerides in Medium VLDL	0.01443012	0.005	0.011	-6.0381054
Free Cholesterol in LDL	-0.0136955	0.003	0.006	-5.427253
Total Concentration of Branched-Chain Amino Acids (Leucine + Isoleucine + Valine)	0.01353676	9.67E-05	3.53E-04	-2.3076526
Phospholipids in Medium HDL	-0.0118299	6.60E-04	0.002	-4.1090173
Free Cholesterol in VLDL	0.01125517	0.014	0.026	-6.9176002
Free Cholesterol in Large LDL	-0.0108641	9.41E-04	0.003	-4.4380271
Degree of Unsaturation	-0.0108078	2.22E-08	4.67E-07	5.7378133
Glycine	-0.0107989	2.97E-04	9.42E-04	-3.3640638
Free Cholesterol in Large HDL	-0.0098638	1.18E-07	1.65E-06	4.1211456
Sphingomyelins	-0.0097976	3.26E-04	9.97E-04	-3.4517626
Glutamine	-0.0097716	0.002	0.004	-4.8884566
Phospholipids in Very Large HDL	-0.0096305	1.81E-05	8.69E-05	-0.7186157
Cholesterol in Chylomicrons and Extremely Large VLDL	0.00932864	7.24E-06	3.80E-05	0.15717425
Cholesterol in Very Large HDL	-0.0084456	1.97E-06	1.38E-05	1.40599482
Triglycerides in Small VLDL	0.00832161	0.004	0.009	-5.7979769
Phospholipids in Large VLDL	0.00812043	8.44E-05	3.22E-04	-2.1796764
Valine	0.00793755	6.21E-05	2.43E-04	-1.8890652
Cholesterol in Large VLDL	0.0078455	0.001	0.003	-4.769888
Phospholipids in Chylomicrons and Extremely Large VLDL	0.00770811	1.11E-06	8.46E-06	1.95798387
Cholesteryl Esters in Very Large HDL	-0.0072612	4.93E-07	4.60E-06	2.73734424
Free Cholesterol in IDL	-0.0072519	0.004	0.009	-5.7646116
Average Diameter for VLDL Particles	0.00713153	4.68E-05	1.87E-04	-1.6219849
Phospholipids in Very Large VLDL	0.00592848	1.64E-05	8.08E-05	-0.6215686
Free Cholesterol in Medium HDL	-0.0058649	7.68E-07	6.56E-06	2.31022898
Cholesterol in Small HDL	-0.0055793	0.024	0.041	-7.345126
Cholesterol in Very Large VLDL	0.0053836	3.36E-04	0.001	-3.4776102

Average Diameter for HDL Particles	-0.0052661	1.40E-07	1.81E-06	3.95507786
Leucine	0.00525664	2.02E-04	6.89E-04	-3.0015308
Cholesteryl Esters in Chylomicrons and Extremely Large VLDL	0.00497362	2.59E-05	1.14E-04	-1.0572697
Free Cholesterol in Large VLDL	0.0049286	2.21E-04	7.15E-04	-3.0876177
Triglycerides in LDL	0.00467411	0.012	0.022	-6.7447195
Free Cholesterol in Chylomicrons and Extremely Large VLDL	0.00465699	2.30E-06	1.54E-05	1.25640448
Free Cholesterol in Medium LDL	-0.0036735	0.024	0.041	-7.3564312
Triglycerides in Small HDL	0.00347097	1.46E-04	5.22E-04	-2.6973656
Pyruvate	0.00337998	0.008	0.017	-6.4400857
Cholesteryl Esters in Large VLDL	0.0033136	0.009	0.018	-6.510032
Isoleucine	0.00322728	9.88E-04	0.003	-4.4827739
Triglycerides in Very Small VLDL	0.0032063	0.003	0.006	-5.3691529
Free Cholesterol in Very Large VLDL	0.0031984	3.76E-05	1.62E-04	-1.4137612
Triglycerides in IDL	0.00301004	0.015	0.028	-6.9781555
Phenylalanine	0.00288889	4.78E-06	2.77E-05	0.55471197
Citrate	-0.0028745	4.60E-05	1.87E-04	-1.6048887
Tyrosine	0.00287117	1.84E-04	6.44E-04	-2.9136795
Triglycerides in Large LDL	0.00272922	0.025	0.043	-7.406246
Cholesteryl Esters in Very Large VLDL	0.00234216	0.003	0.007	-5.5594145
Free Cholesterol in Small HDL	-0.002118	0.011	0.020	-6.6422627
Free Cholesterol in Very Large HDL	-0.0014708	3.81E-04	0.001	-3.5970292
Triglycerides in Medium LDL	0.00138276	0.008	0.017	-6.4198263
Triglycerides in Small LDL	9.77E-04	0.001	0.003	-4.5323666
Total Concentration of Lipoprotein Particles	-6.16E-04	6.64E-06	3.72E-05	0.23884832
Concentration of HDL Particles	-6.13E-04	2.81E-06	1.81E-05	1.06470725
Concentration of Large HDL Particles	-2.34E-04	1.61E-08	3.85E-07	6.05324159
Concentration of Medium HDL Particles	-2.29E-04	2.94E-06	1.83E-05	1.01906074
Concentration of Very Large HDL Particles	-2.52E-05	4.04E-05	1.70E-04	-1.4813689
Total Lipids in Large HDL	-0.0550021	8.77E-09	2.46E-07	6.64098703
Triglycerides in VLDL	0.0517833	9.13E-05	3.41E-04	-2.2533645
Total Triglycerides	0.04814629	2.19E-04	7.15E-04	-3.0766331
Total Lipids in VLDL	0.04800426	0.002	0.004	-4.9025605
HDL Cholesterol	-0.0435255	6.34E-10	5.32E-08	9.20071015
Total Lipids in HDL	-0.0422232	9.74E-08	1.49E-06	4.30564083
Total Lipids in Chylomicrons and Extremely Large VLDL	0.04050946	8.40E-07	6.72E-06	2.22400265
Monounsaturated Fatty Acids	0.03997764	3.20E-04	9.95E-04	-3.4322284
Cholesteryl Esters in HDL	-0.0396672	4.36E-10	5.32E-08	9.56600928

Cholesterol in Large HDL	-0.0381858	5.00E-09	1.68E-07	7.18702667
Cholesteryl Esters in Large HDL	-0.0320867	3.32E-09	1.39E-07	7.58760746
Total Esterified Cholesterol	-0.0318687	2.05E-04	6.89E-04	-3.0153843
Total Cholesterol	-0.0316696	6.50E-04	0.002	-4.0950585
Phospholipids in Large HDL	-0.0309497	6.94E-08	1.17E-06	4.63380907
Albumin	-0.0303164	1.75E-09	9.79E-08	8.21078286
Triglycerides in Chylomicrons and Extremely Large VLDL	0.02878337	7.81E-07	6.56E-06	2.29512139
Phospholipids in HDL	-0.0286314	4.37E-06	2.62E-05	0.64043592
Glucose	0.0286044	3.45E-04	0.001	-3.5042296
Clinical LDL Cholesterol	-0.0269427	0.022	0.038	-7.2739963
Total Lipids in Large VLDL	0.02627349	3.70E-04	0.001	-3.5682994
Apolipoprotein A1	-0.0257832	3.45E-07	3.41E-06	3.08191499
Total Lipids in Very Large VLDL	0.02556952	1.89E-05	8.84E-05	-0.7613633
Total Lipids in Medium HDL	-0.0238258	2.28E-05	1.03E-04	-0.9363499
Glucose-lactate	0.02242116	0.004	0.009	-5.8489763
Cholesterol in Medium HDL	-0.0220813	1.60E-07	1.81E-06	3.82219704
Cholesterol in Large LDL	-0.0209923	0.005	0.010	-5.927213
Omega-3 Fatty Acids	-0.0207275	0.007	0.015	-6.3163827
Total Lipids in Large LDL	-0.0204324	0.013	0.023	-6.7907941
Phosphatidylcholines	-0.0201903	0.002	0.004	-5.0808438
Total Cholines	-0.0201385	0.001	0.003	-4.7135232
Cholesterol in IDL	-0.0201196	0.001	0.004	-4.8192508
Phospholipids in VLDL	0.01973679	0.003	0.007	-5.6153282
Cholesteryl Esters in Medium HDL	-0.0189483	1.62E-07	1.81E-06	3.81531299
Total Lipids in IDL	-0.018779	0.008	0.015	-6.3383806
Glycoprotein Acetyls	0.01843316	3.10E-07	3.25E-06	3.18676691
Docosahexaenoic Acid	-0.0175211	1.46E-06	1.06E-05	1.69536984
Triglycerides in Very Large VLDL	0.01731662	1.33E-05	6.79E-05	-0.4264812
Cholesteryl Esters in IDL	-0.0168277	0.002	0.004	-4.8747049
Phosphoglycerides	-0.0168033	0.010	0.020	-6.6163926
Total Phospholipids in Lipoprotein Particles	-0.0166588	0.010	0.020	-6.6114267
Total Lipids in Very Large HDL	-0.0164213	7.08E-06	3.80E-05	0.17759913
Cholesteryl Esters in Large LDL	-0.0160292	0.012	0.022	-6.7406992
Free Cholesterol in HDL	-0.0159914	4.01E-08	7.48E-07	5.16550933
Total Free Cholesterol	-0.0158511	0.016	0.028	-6.9932024
Triglycerides in Large VLDL	0.01537303	6.48E-04	0.002	-4.0911376
Triglycerides in Medium VLDL	0.01443012	0.005	0.011	-6.0381054

Free Cholesterol in LDL	-0.0136955	0.003	0.006	-5.427253
Total Concentration of Branched-Chain Amino Acids (Leucine + Isoleucine + Valine)	0.01353676	9.67E-05	3.53E-04	-2.3076526
Phospholipids in Medium HDL	-0.0118299	6.60E-04	0.002	-4.1090173
Free Cholesterol in VLDL	0.01125517	0.014	0.026	-6.9176002
Free Cholesterol in Large LDL	-0.0108641	9.41E-04	0.003	-4.4380271
Degree of Unsaturation	-0.0108078	2.22E-08	4.67E-07	5.7378133
Glycine	-0.0107989	2.97E-04	9.42E-04	-3.3640638
Free Cholesterol in Large HDL	-0.0098638	1.18E-07	1.65E-06	4.1211456
Sphingomyelins	-0.0097976	3.26E-04	9.97E-04	-3.4517626
Glutamine	-0.0097716	0.002	0.004	-4.8884566
Phospholipids in Very Large HDL	-0.0096305	1.81E-05	8.69E-05	-0.7186157
Cholesterol in Chylomicrons and Extremely Large VLDL	0.00932864	7.24E-06	3.80E-05	0.15717425
Cholesterol in Very Large HDL	-0.0084456	1.97E-06	1.38E-05	1.40599482
Triglycerides in Small VLDL	0.00832161	0.004	0.009	-5.7979769
Phospholipids in Large VLDL	0.00812043	8.44E-05	3.22E-04	-2.1796764
Valine	0.00793755	6.21E-05	2.43E-04	-1.8890652
Cholesterol in Large VLDL	0.0078455	0.001	0.003	-4.769888
Phospholipids in Chylomicrons and Extremely Large VLDL	0.00770811	1.11E-06	8.46E-06	1.95798387
Cholesteryl Esters in Very Large HDL	-0.0072612	4.93E-07	4.60E-06	2.73734424
Free Cholesterol in IDL	-0.0072519	0.004	0.009	-5.7646116
Average Diameter for VLDL Particles	0.00713153	4.68E-05	1.87E-04	-1.6219849
Phospholipids in Very Large VLDL	0.00592848	1.64E-05	8.08E-05	-0.6215686
Free Cholesterol in Medium HDL	-0.0058649	7.68E-07	6.56E-06	2.31022898
Cholesterol in Small HDL	-0.0055793	0.024	0.041	-7.345126
Cholesterol in Very Large VLDL	0.0053836	3.36E-04	0.001	-3.4776102
Triglycerides in IDL	0.00301004	1.40E-07	1.81E-06	-6.9781555
Phenylalanine	0.00288889	2.02E-04	6.89E-04	0.55471197
Citrate	-0.0028745	2.59E-05	1.14E-04	-1.6048887
Tyrosine	0.00287117	2.21E-04	7.15E-04	-2.9136795
Triglycerides in Large LDL	0.00272922	0.012	0.022	-7.406246
Cholesteryl Esters in Very Large VLDL	0.00234216	2.30E-06	1.54E-05	-5.5594145
Free Cholesterol in Small HDL	-0.002118	0.024	0.041	-6.6422627
Free Cholesterol in Very Large HDL	-0.0014708	1.46E-04	5.22E-04	-3.5970292
Triglycerides in Medium LDL	0.00138276	0.008	0.017	-6.4198263
Triglycerides in Small LDL	9.77E-04	0.009	0.018	-4.5323666
Total Concentration of Lipoprotein Particles	-6.16E-04	9.88E-04	0.003	0.23884832
Concentration of HDL Particles	-6.13E-04	0.003	0.006	1.06470725

Concentration of Large HDL Particles	-2.34E-04	3.76E-05	1.62E-04	6.05324159
Concentration of Medium HDL Particles	-2.29E-04	0.015	0.028	1.01906074
Concentration of Very Large HDL Particles	-2.52E-05	4.78E-06	2.77E-05	-1.4813689

Positive log fold-change (log2FC) indicates higher levels in the Low HGS/BMI only group. FDR adjusted *P* values calculated using Benjamini–Hochberg correction. Linear modelling was adjusted for age, sex, ethnicity, education, deprivation index, protein intake, physical activity, smoking, alcohol use, diabetes, hypertension, coronary heart disease, and chronic kidney disease.

Abbreviations: FDR, false discovery rate; FC, fold change; HDL, high-density lipoprotein; IDL, intermediate-density lipoprotein; LDL, low-density lipoprotein; VLDL, very-low-density lipoprotein.

Table S13. Significant Associations Between Differential Metabolites(adjusted *P* < 0.01) and Incident Heart Failure Among Participants with **Confirmed Sarcopenia** vs **Reference** Groups

**Metabolite**	**Category**	**HR (95% CI)**	**Adjusted *P***
Histidine	Amino acids	0.92 (0.90–0.94)	2.15E-11
Tyrosine		1.08 (1.06–1.11)	2.9E-11
Phenylalanine		1.05 (1.03–1.07)	1.54E-06
Glycine		0.94 (0.91–0.96)	2.72E-05
Glutamine		0.96 (0.94–0.98)	0.003
Glucose-lactate	Carbohydrates (glycolysis/TCA)	1.08 (1.06–1.11)	1E-11
Glucose		1.08 (1.06–1.10)	3.3E-10
Cholesterol in Small HDL	Cholesterol	0.91 (0.89–0.93)	5.46E-13
LDL Cholesterol		0.94 (0.92–0.96)	6.73E-06
Cholesterol in Large LDL		0.94 (0.92–0.96)	1.56E-05
Clinical LDL Cholesterol		0.95 (0.93–0.97)	1.85E-04
Total Cholesterol		0.96 (0.93–0.98)	0.019
Cholesteryl Esters in Small HDL	Cholesteryl esters	0.90 (0.88–0.92)	2.77E-16
Cholesteryl Esters in Large LDL		0.94 (0.92–0.96)	1.01E-05
Total Esterified Cholesterol		0.95 (0.93–0.98)	0.005
Cholesteryl Esters in Medium HDL		0.95 (0.93–0.98)	0.028
Degree of Unsaturation	Fatty acids	0.90 (0.89–0.92)	3.23E-23
Docosahexaenoic Acid		0.89 (0.87–0.91)	8.15E-20
Omega-3 Fatty Acids		0.90 (0.88–0.92)	2.12E-16
Linoleic Acid		0.95 (0.93–0.97)	4.13E-04
Free Cholesterol in Medium LDL	Free cholesterol	0.93 (0.91–0.95)	1.28E-07
Free Cholesterol in Small LDL		0.94 (0.92–0.96)	2.17E-06
Free Cholesterol in LDL		0.94 (0.92–0.96)	1.33E-05
Free Cholesterol in Very Large HDL		1.06 (1.04–1.09)	3.85E-05
Free Cholesterol in Large LDL		0.95 (0.92–0.97)	2.79E-04
3-Hydroxybutyrate	Ketone bodies	1.07 (1.05–1.09)	1.65E-08
Phospholipids in Large LDL	Phospholipids	0.95 (0.93–0.98)	0.007
Phospholipids in Very Large HDL		1.05 (1.03–1.08)	0.009
Phospholipids in Small HDL		0.96 (0.94–0.98)	0.044
Albumin	Proteins Others / Fluid balance	0.92 (0.91–0.94)	1.26E-42
Glycoprotein Acetyls		1.13 (1.10–1.15)	6.38E-23
Triglycerides in IDL	Triglycerides	1.08 (1.06–1.10)	1.37E-10
Triglycerides in Large LDL		1.07 (1.05–1.09)	2.36E-08
Triglycerides in Very Small VLDL		1.06 (1.04–1.08)	1.14E-05
Triglycerides in LDL		1.06 (1.04–1.08)	1.51E-05
Triglycerides in Large HDL		1.06 (1.04–1.08)	4.19E-05
Triglycerides in Medium LDL		1.04 (1.02–1.07)	0.006
Triglycerides in HDL		1.04 (1.02–1.07)	0.007
Triglycerides in Medium HDL		1.04 (1.02–1.07)	0.01

Cox proportional hazards regression was performed for metabolites with FDR-adjusted *P* value < 0.01, identified between individuals with confirmed sarcopenia (low HGS and low ALM per 2014 FNIH criteria) and reference group.

HRs and 95% CIs represent the risk of incident heart failure per 1-SD increase in $\log_{10}$-transformed, standardised plasma metabolite concentrations. Models were adjusted for age, sex, ethnicity, education level, socioeconomic deprivation, protein intake, physical activity, smoking, alcohol use, diabetes, hypertension, coronary heart disease, and chronic kidney disease.

P values were corrected for multiple comparisons using the Bonferroni method.

Abbreviations: ALM, appendicular lean mass; CI, confidence interval; FDR, false discovery rate; FNIH, Foundation for the National Institutes of Health; HDL, high-density lipoprotein; HF, heart failure; HGS, handgrip strength; HR, hazard ratio; IDL, intermediate-density lipoprotein; LDL, low-density lipoprotein; SD, standard deviation; TCA, tricarboxylic acid; VLDL, very-low-density lipoprotein.

Table S14. Significant Associations of differential metabolites (FDR < 0.05 and |log₂ fold change| > 0.02) of sarcopenia phenotypes with incident heart failure.

Metabolite	Category	HR (95%CI)	Adjusted *P* value
**Confirmed Sarcopenia vs. Reference**			
Glutamine	Amino acids	0.96 (0.94–0.98)	8.23E-04
Glycine		0.94 (0.91–0.96)	6.79E-06
Glucose	Carbohydrates (glycolysis/TCA)	1.08 (1.06–1.10)	8.25E-11
Glucose-lactate		1.08 (1.06–1.11)	2.50E-12
HDL Cholesterol	Cholesterol	0.96 (0.94–0.99)	0.112
Total Cholesterol		0.96 (0.93–0.98)	0.005
Clinical LDL Cholesterol		0.95 (0.93–0.97)	4.64E-05
Cholesteryl Esters in HDL	Cholesteryl esters	0.95 (0.93–0.98)	0.013
Total Esterified Cholesterol		0.95 (0.93–0.98)	0.001
Omega-3 Fatty Acids	Fatty acids	0.90 (0.88–0.92)	5.29E-17
Docosahexaenoic Acid		0.89 (0.87–0.91)	2.04E-20
Albumin	Proteins Others / Fluid balance	0.92 (0.91–0.94)	3.14E-43
Glycoprotein Acetyls		1.13 (1.10–1.15)	1.59E-23
**Low HGS/BMI only vs. Reference**			
HDL Cholesterol	Cholesterol	0.96 (0.94–0.98)	0.020
Cholesteryl Esters in HDL	Cholesteryl esters	0.95 (0.93–0.98)	0.002
Total Esterified Cholesterol		0.95 (0.93–0.97)	3.73E-05
Albumin	Proteins Others / Fluid balance	0.92 (0.91–0.93)	2.10E-56
Glycoprotein Acetyls		1.13 (1.11–1.15)	8.09E-29
Triglycerides in Large VLDL	Triglycerides	0.97 (0.95–0.99)	0.014
Triglycerides in Medium VLDL		0.96 (0.94–0.98)	0.004

Cox proportional hazards regression models were performed for each metabolite identified as both FDR < 0.05 and |log₂ fold change| > 0.02 in the a) confirmed sarcopenia vs. Reference and b) low HGS/BMI only vs. Reference comparisons. Baseline metabolite levels were standardized (log_{10}-transformed and z-scored), and hazard ratios (HRs) reflect the relative risk per 1-SD increase in metabolite concentration. Models were adjusted for age, sex, ethnicity, education level, deprivation index, protein intake, physical activity, smoking, alcohol use, diabetes, hypertension, coronary heart disease, and chronic kidney disease. *P* values were Bonferroni-corrected for multiple comparisons.

Abbreviations: BMI, body mass index; CI, confidence interval; FDR, false discovery rate; HDL, high-density lipoprotein; HF, heart failure; HGS, handgrip strength; HR, hazard ratio; LDL, low-density lipoprotein; SD, standard deviation; TCA, tricarboxylic acid; VLDL, very-low-density lipoprotein.

Table S15. Metabolites selected by LASSO regression from Confirmed Sarcopenia and Low HGS/BMI groups (vs. Reference group respectively).

Group	Selected Metabolites
**$\lvert log_2FC \rvert > 0.02$**	
6 metabolites	Albumin, Omega-3 Fatty Acids, Clinical LDL Cholesterol, Glycoprotein Acetyls, Glycine, Glucose-lactate
4 metabolites	Albumin, Triglycerides in Medium VLDL, Glycoprotein Acetyls, Cholesteryl Esters in HDL
**FDR < 0.01**	
13 metabolites	Albumin, Degree of Unsaturation, Glycoprotein Acetyls, Omega-3 Fatty Acids, Histidine, Tyrosine, 3-Hydroxybutyrate, Linoleic Acid, Glycine, Free Cholesterol in Very Large HDL, Triglycerides in Medium LDL, Phenylalanine, Triglycerides in IDL
14 metabolites	Albumin, Glycoprotein Acetyls, Omega-3 Fatty Acids, Histidine, Tyrosine, Phenylalanine, Creatinine, Glycine, Linoleic Acid, Leucine, Citrate, Triglycerides in Large LDL, Polyunsaturated Fatty Acids, Glucose-lactate

Abbreviations: BMI, body mass index; FC, fold change; FDR, false discovery rate; HDL, high-density lipoprotein; HGS, handgrip strength; IDL, intermediate-density lipoprotein; LASSO, least absolute shrinkage and selection operator; LDL, low-density lipoprotein; VLDL, very-low-density lipoprotein.

Table S16. Mediation analysis of LASSO-selected metabolites in the associations between sarcopenia phenotypes and incident heart failure.

Groups	Mediators	N	Total effect	Natural direct effect	Natural indirect effect	PM
			HR (95%CI)	HR (95%CI)	HR (95%CI)	
Confirmed Sarcopenia Vs. Both Normal	3-Hydroxybutyrate	245, 851	1.597 (1.365–1.821) ***	1.597 (1.365–1.821) ***	1.008 (1.005–1.013) ***	2.20%
	Albumin		1.606 (1.373–1.834) ***	1.606 (1.373–1.834) ***	1.027 (1.023–1.033) ***	7.08%
	Degree of Unsaturation		1.607 (1.371–1.827) ***	1.607 (1.371–1.827) ***	1.028 (1.022–1.034) ***	7.31%
	Free Cholesterol in Very Large HDL		1.593 (1.361–1.820) ***	1.593 (1.361–1.820) ***	0.988 (0.983–0.993) ***	-3.36%
	Glycine		1.604 (1.372–1.827) ***	1.604 (1.372–1.827) ***	1.017 (1.010–1.025) ***	4.48%
	Glycoprotein Acetyls		1.616 (1.387–1.839) ***	1.616 (1.387–1.839) ***	1.058 (1.046–1.072) ***	14.49%
	Histidine		1.602 (1.370–1.825) ***	1.602 (1.370–1.825) ***	1.012 (1.008–1.018) ***	3.27%
	Linoleic Acid		1.601 (1.368–1.826) ***	1.601 (1.368–1.826) ***	1.003 (1.001–1.006) **	0.88%
	Omega-3 Fatty Acids		1.611 (1.377–1.832) ***	1.611 (1.377–1.832) ***	1.020 (1.014–1.027) ***	5.12%
	Phenylalanine		1.597 (1.364–1.823) ***	1.597 (1.364–1.823) ***	1.010 (1.006–1.014) ***	2.62%
	Triglycerides in IDL		1.600 (1.370–1.825) ***	1.600 (1.370–1.825) ***	1.010 (1.007–1.015) ***	2.74%
	Triglycerides in Medium LDL		1.599 (1.369–1.825) ***	1.599 (1.369–1.825) ***	1.007 (1.003–1.011) ***	1.85%
	Tyrosine		1.601 (1.366–1.830) ***	1.601 (1.366–1.830) ***	1.020 (1.013–1.027) ***	5.14%
Low HGS/BMI only vs. Both Normal	Albumin	262, 654	1.760 (1.668–1.853) ***	1.727 (1.639–1.818) ***	1.019 (1.016–1.022) ***	4.27%
	Citrate		1.751 (1.660–1.843) ***	1.762 (1.670–1.857) ***	0.994 (0.992–0.997) ***	-1.37%
	Creatinine		1.750 (1.659–1.843) ***	1.748 (1.658–1.840) ***	1.001 (1.000–1.002) *	0.25%
	Glucose-lactate		1.753 (1.663–1.845) ***	1.742 (1.653–1.835) ***	1.006 (1.004–1.008) ***	1.35%
	Glycine		1.758 (1.664–1.850) ***	1.740 (1.650–1.830) ***	1.010 (1.006–1.014) ***	2.30%
	Glycoprotein Acetyls		1.770 (1.678–1.864) ***	1.704 (1.617–1.795) ***	1.039 (1.032–1.046) ***	8.55%
	Histidine		1.757 (1.665–1.848) ***	1.746 (1.656–1.837) ***	1.006 (1.004–1.008) ***	1.48%
	Leucine		1.751 (1.660–1.842) ***	1.758 (1.665–1.851) ***	0.996 (0.995–0.999) ***	-0.82%
	Linoleic Acid		1.753 (1.660–1.844) ***	1.750 (1.658–1.842) ***	1.001 (1.001–1.002) ***	0.32%
	Omega-3 Fatty Acids		1.755 (1.663–1.847) ***	1.741 (1.649–1.833) ***	1.008 (1.006–1.011) ***	1.87%
	Phenylalanine		1.754 (1.662–1.846) ***	1.741 (1.650–1.832) ***	1.008 (1.005–1.010) ***	1.74%
	Polyunsaturated Fatty Acids		1.752 (1.660–1.844) ***	1.750 (1.658–1.842) ***	1.002 (1.001–1.003) ***	0.36%
	Triglycerides in Large LDL		1.759 (1.666–1.851) ***	1.737 (1.647–1.825) ***	1.012 (1.008–1.016) ***	2.84%
	Tyrosine		1.758 (1.667–1.851) ***	1.738 (1.646–1.830) ***	1.011 (1.008–1.015) ***	2.61%

Adjusted for full sets of metabolites. PM represents the proportion of the total effect mediated through the metabolite.

[*] $P < 0.05$; [**] $P < 0.01$; [***] $P < 0.001$.

Abbreviations: BMI, body mass index; CI, confidence interval; HDL, high-density lipoprotein; HGS, handgrip strength; HR, hazard ratio; IDL, intermediate-density lipoprotein; LASSO, least absolute shrinkage and selection operator; LDL, low-density lipoprotein; PM, proportion mediated.

Figure S1. Flowchart of participant selection of the UK Biobank study.

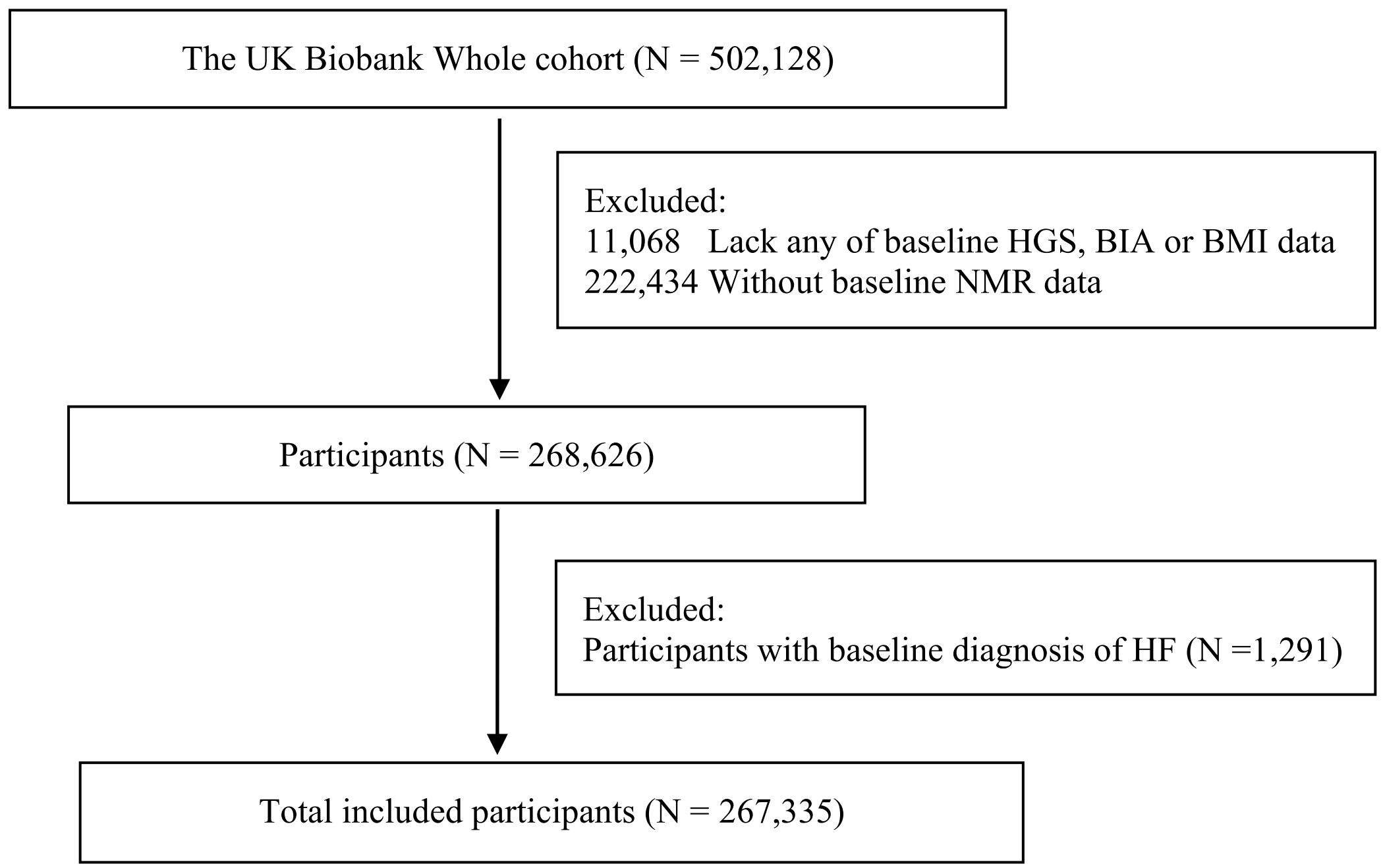

HGS, handgrip strength; BIA, Bioelectrical impedance analysis; BMI, body mass index; HF, heart failure; NMR, Nuclear Magnetic Resonance.

Figure S2. Marginal predicted risk of incident heart failure across sarcopenia phenotypes by age and sex.

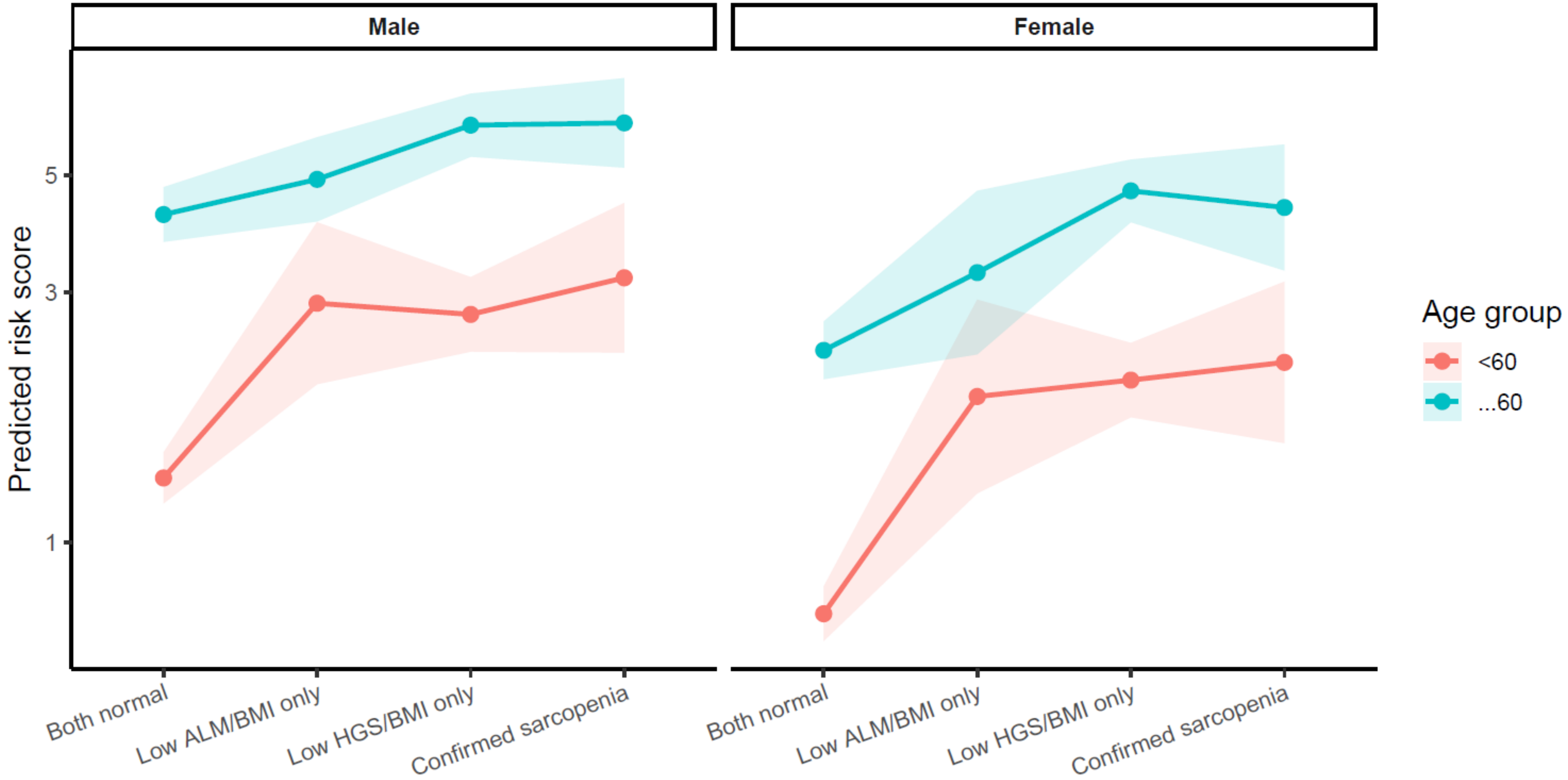

Estimates were derived from Cox models including interaction terms between sarcopenia phenotypes and age and sex (Model 3 + sarco4_group × Age_group + sarco4_group × Sex_group). Shaded areas represent 95% confidence intervals.
Abbreviations: BMI, body mass index; ALM, appendicular lean mass; HGS, handgrip strength.

Figure S3. Nonlinear Associations of EWGSOP2-defined Indicators with Risk of Incident Heart Failure

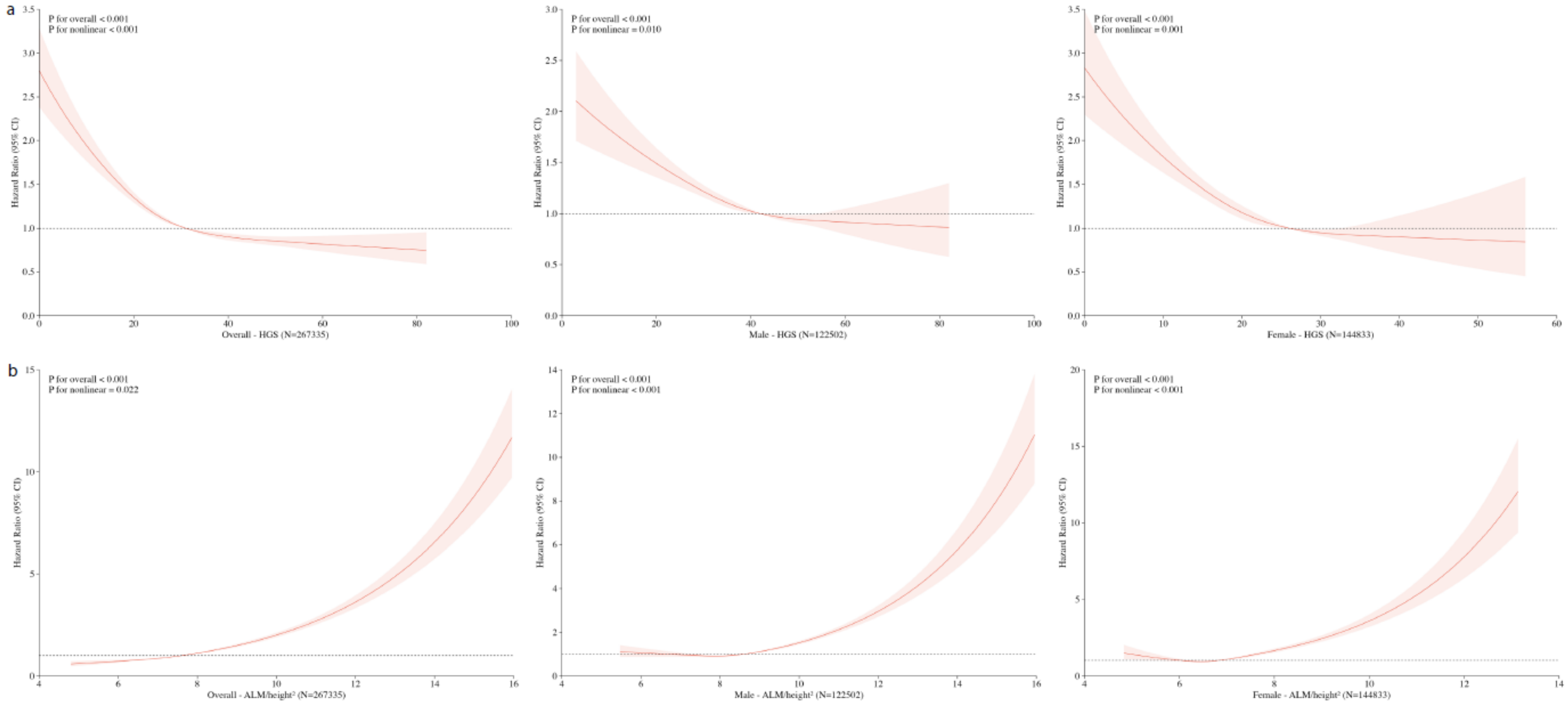

Restricted cubic spline models showing the nonlinear associations between absolute handgrip strength (HGS, kg; a) and appendicular lean mass indexed to height squared (ALM/height²; b) with incident heart failure. Models were fully adjusted for demographic, behavioural, and clinical covariates. The red line denotes the adjusted hazard ratio (HR) and the shaded area the 95% confidence interval. Knots were placed at the 5th, 35th, 65th, and 95th percentiles, with the median value used as the reference point. ALM, appendicular lean mass; HGS, handgrip strength; EWGSOP2, European Working Group on Sarcopenia in Older People 2.

Figure S4. Subgroup Analyses of the associations between FNIH-defined Sarcopenia Indicators with incident heart failure in segmented Cox regression.

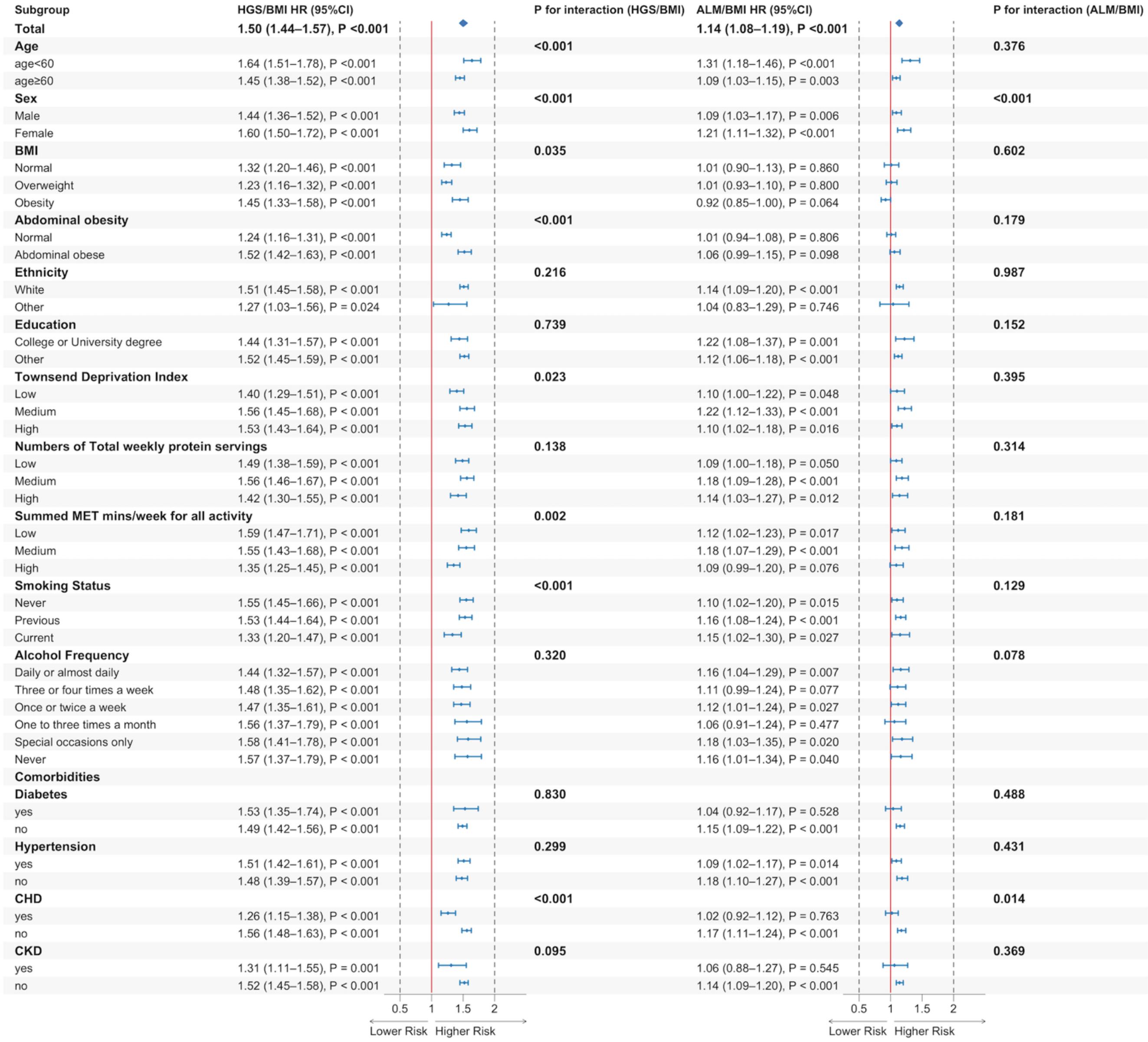

Subgroup	HGS/BMI HR (95%CI)	P for interaction (HGS/BMI)	ALM/BMI HR (95%CI)	P for interaction (ALM/BMI)
**Total**	**1.50 (1.44–1.57), P <0.001**		**1.14 (1.08–1.19), P <0.001**	
**Age**		**<0.001**		**0.376**
age<60	1.64 (1.51–1.78), P <0.001		1.31 (1.18–1.46), P <0.001	
age≥60	1.45 (1.38–1.52), P <0.001		1.09 (1.03–1.15), P = 0.003	
**Sex**		**<0.001**		**<0.001**
Male	1.44 (1.36–1.52), P < 0.001		1.09 (1.03–1.17), P = 0.006	
Female	1.60 (1.50–1.72), P < 0.001		1.21 (1.11–1.32), P <0.001	
**BMI**		**0.035**		**0.602**
Normal	1.32 (1.20–1.46), P <0.001		1.01 (0.90–1.13), P = 0.860	
Overweight	1.23 (1.16–1.32), P <0.001		1.01 (0.93–1.10), P = 0.800	
Obesity	1.45 (1.33–1.58), P <0.001		0.92 (0.85–1.00), P = 0.064	
**Abdominal obesity**		**<0.001**		**0.179**
Normal	1.24 (1.16–1.31), P <0.001		1.01 (0.94–1.08), P = 0.806	
Abdominal obese	1.52 (1.42–1.63), P <0.001		1.06 (0.99–1.15), P = 0.098	
**Ethnicity**		**0.216**		**0.987**
White	1.51 (1.45–1.58), P < 0.001		1.14 (1.09–1.20), P < 0.001	
Other	1.27 (1.03–1.56), P = 0.024		1.04 (0.83–1.29), P = 0.746	
**Education**		**0.739**		**0.152**
College or University degree	1.44 (1.31–1.57), P < 0.001		1.22 (1.08–1.37), P = 0.001	
Other	1.52 (1.45–1.59), P < 0.001		1.12 (1.06–1.18), P < 0.001	
**Townsend Deprivation Index**		**0.023**		**0.395**
Low	1.40 (1.29–1.51), P < 0.001		1.10 (1.00–1.22), P = 0.048	
Medium	1.56 (1.45–1.68), P < 0.001		1.22 (1.12–1.33), P < 0.001	
High	1.53 (1.43–1.64), P < 0.001		1.10 (1.02–1.18), P = 0.016	
**Numbers of Total weekly protein servings**		**0.138**		**0.314**
Low	1.49 (1.38–1.59), P < 0.001		1.09 (1.00–1.18), P = 0.050	
Medium	1.56 (1.46–1.67), P < 0.001		1.18 (1.09–1.28), P < 0.001	
High	1.42 (1.30–1.55), P < 0.001		1.14 (1.03–1.27), P = 0.012	
**Summed MET mins/week for all activity**		**0.002**		**0.181**
Low	1.59 (1.47–1.71), P < 0.001		1.12 (1.02–1.23), P = 0.017	
Medium	1.55 (1.43–1.68), P < 0.001		1.18 (1.07–1.29), P < 0.001	
High	1.35 (1.25–1.45), P < 0.001		1.09 (0.99–1.20), P = 0.076	
**Smoking Status**		**<0.001**		**0.129**
Never	1.55 (1.45–1.66), P < 0.001		1.10 (1.02–1.20), P = 0.015	
Previous	1.53 (1.44–1.64), P < 0.001		1.16 (1.08–1.24), P < 0.001	
Current	1.33 (1.20–1.47), P < 0.001		1.15 (1.02–1.30), P = 0.027	
**Alcohol Frequency**		**0.320**		**0.078**
Daily or almost daily	1.44 (1.32–1.57), P < 0.001		1.16 (1.04–1.29), P = 0.007	
Three or four times a week	1.48 (1.35–1.62), P < 0.001		1.11 (0.99–1.24), P = 0.077	
Once or twice a week	1.47 (1.35–1.61), P < 0.001		1.12 (1.01–1.24), P = 0.027	
One to three times a month	1.56 (1.37–1.79), P < 0.001		1.06 (0.91–1.24), P = 0.477	
Special occasions only	1.58 (1.41–1.78), P < 0.001		1.18 (1.03–1.35), P = 0.020	
Never	1.57 (1.37–1.79), P < 0.001		1.16 (1.01–1.34), P = 0.040	
**Comorbidities**				
**Diabetes**		**0.830**		**0.488**
yes	1.53 (1.35–1.74), P < 0.001		1.04 (0.92–1.17), P = 0.528	
no	1.49 (1.42–1.56), P < 0.001		1.15 (1.09–1.22), P < 0.001	
**Hypertension**		**0.299**		**0.431**
yes	1.51 (1.42–1.61), P < 0.001		1.09 (1.02–1.17), P = 0.014	
no	1.48 (1.39–1.57), P < 0.001		1.18 (1.10–1.27), P < 0.001	
**CHD**		**<0.001**		**0.014**
yes	1.26 (1.15–1.38), P < 0.001		1.02 (0.92–1.12), P = 0.763	
no	1.56 (1.48–1.63), P < 0.001		1.17 (1.11–1.24), P < 0.001	
**CKD**		**0.095**		**0.369**
yes	1.31 (1.11–1.55), P = 0.001		1.06 (0.88–1.27), P = 0.545	
no	1.52 (1.45–1.58), P < 0.001		1.14 (1.09–1.20), P < 0.001	

Forest plots show HRs (95% CIs) for “Lower” vs “Higher” HGS/BMI and ALM/BMI (FNIH) for associations with incident HF, based on segmented Cox regression with sex-specific inflection points (HGS/BMI: 1.48 [males], 0.82 [females]; ALM/BMI: 0.85 [males], 0.72 [females]). The “Higher” group served as the reference. Red vertical lines indicate the reference (HR = 1) at the inflection point. *P* for interaction was obtained from models including subgroup × sarcopenia indicator terms.

Abbreviations: HR, hazard ratio; CI, confidence interval; HGS, handgrip strength; ALM, appendicular lean mass; FNIH, Foundation for the National Institutes of Health; BMI, body mass index.

Figure S5. Stratified associations of absolute handgrip strength (HGS) and relative ALM (ALM/height²) with incident heart failure based on EWGSOP2 criteria in segmented Cox regression.

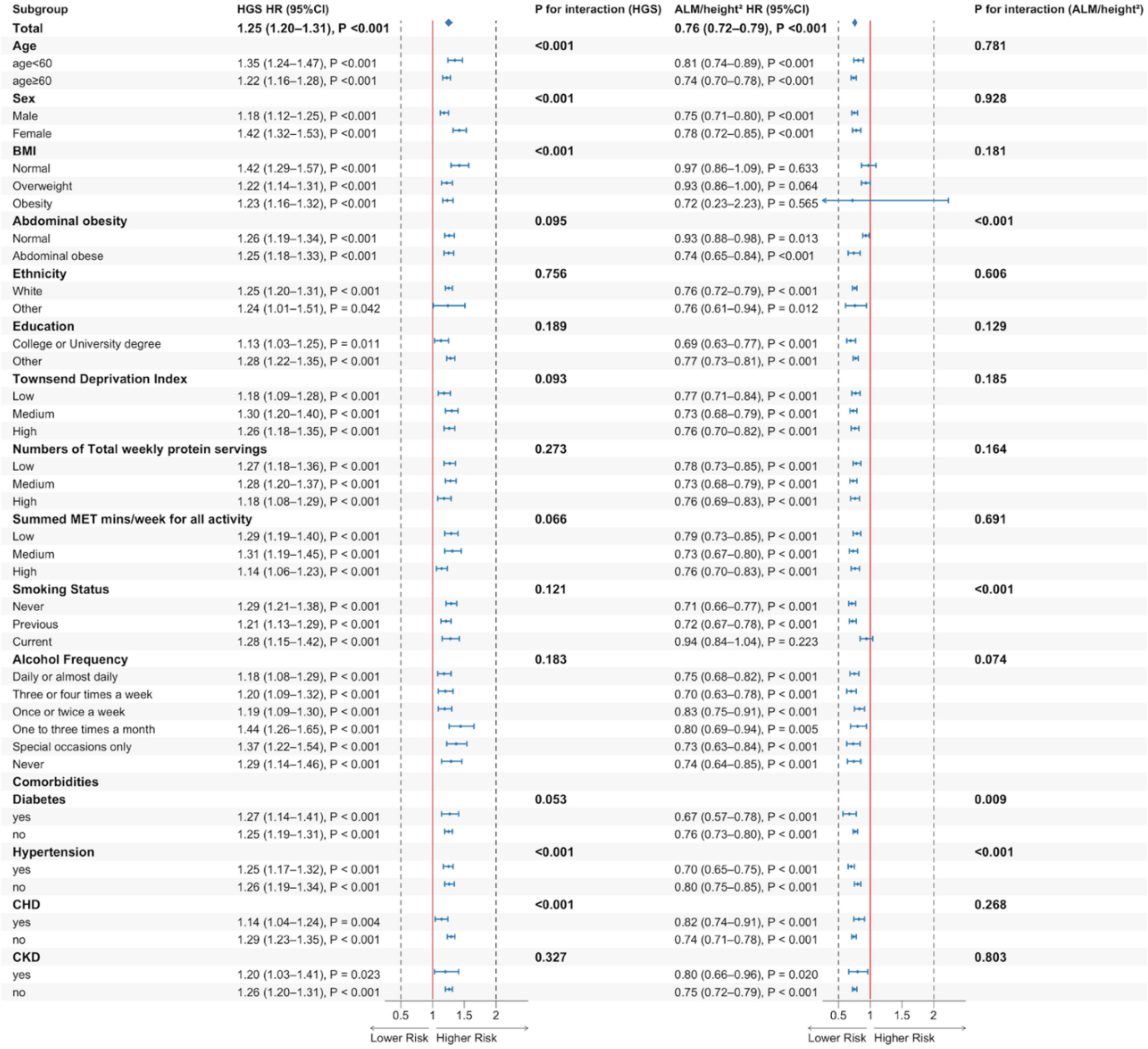

Subgroup	HGS HR (95%CI)	P for interaction (HGS)	ALM/height² HR (95%CI)	P for interaction (ALM/height²)
**Total**	**1.25 (1.20–1.31), P <0.001**		**0.76 (0.72–0.79), P <0.001**	
**Age**		**<0.001**		**0.781**
age<60	1.35 (1.24–1.47), P <0.001		0.81 (0.74–0.89), P <0.001	
age≥60	1.22 (1.16–1.28), P <0.001		0.74 (0.70–0.78), P <0.001	
**Sex**		**<0.001**		**0.928**
Male	1.18 (1.12–1.25), P <0.001		0.75 (0.71–0.80), P <0.001	
Female	1.42 (1.32–1.53), P <0.001		0.78 (0.72–0.85), P <0.001	
**BMI**		**<0.001**		**0.181**
Normal	1.42 (1.29–1.57), P <0.001		0.97 (0.86–1.09), P = 0.633	
Overweight	1.22 (1.14–1.31), P <0.001		0.93 (0.86–1.00), P = 0.064	
Obesity	1.23 (1.16–1.32), P <0.001		0.72 (0.23–2.23), P = 0.565	
**Abdominal obesity**		**0.095**		**<0.001**
Normal	1.26 (1.19–1.34), P <0.001		0.93 (0.88–0.98), P = 0.013	
Abdominal obese	1.25 (1.18–1.33), P <0.001		0.74 (0.65–0.84), P <0.001	
**Ethnicity**		**0.756**		**0.606**
White	1.25 (1.20–1.31), P < 0.001		0.76 (0.72–0.79), P < 0.001	
Other	1.24 (1.01–1.51), P = 0.042		0.76 (0.61–0.94), P = 0.012	
**Education**		**0.189**		**0.129**
College or University degree	1.13 (1.03–1.25), P = 0.011		0.69 (0.63–0.77), P < 0.001	
Other	1.28 (1.22–1.35), P < 0.001		0.77 (0.73–0.81), P < 0.001	
**Townsend Deprivation Index**		**0.093**		**0.185**
Low	1.18 (1.09–1.28), P < 0.001		0.77 (0.71–0.84), P < 0.001	
Medium	1.30 (1.20–1.40), P < 0.001		0.73 (0.68–0.79), P < 0.001	
High	1.26 (1.18–1.35), P < 0.001		0.76 (0.70–0.82), P < 0.001	
**Numbers of Total weekly protein servings**		**0.273**		**0.164**
Low	1.27 (1.18–1.36), P < 0.001		0.78 (0.73–0.85), P < 0.001	
Medium	1.28 (1.20–1.37), P < 0.001		0.73 (0.68–0.79), P < 0.001	
High	1.18 (1.08–1.29), P < 0.001		0.76 (0.69–0.83), P < 0.001	
**Summed MET mins/week for all activity**		**0.066**		**0.691**
Low	1.29 (1.19–1.40), P < 0.001		0.79 (0.73–0.85), P < 0.001	
Medium	1.31 (1.19–1.45), P < 0.001		0.73 (0.67–0.80), P < 0.001	
High	1.14 (1.06–1.23), P < 0.001		0.76 (0.70–0.83), P < 0.001	
**Smoking Status**		**0.121**		**<0.001**
Never	1.29 (1.21–1.38), P < 0.001		0.71 (0.66–0.77), P < 0.001	
Previous	1.21 (1.13–1.29), P < 0.001		0.72 (0.67–0.78), P < 0.001	
Current	1.28 (1.15–1.42), P < 0.001		0.94 (0.84–1.04), P = 0.223	
**Alcohol Frequency**		**0.183**		**0.074**
Daily or almost daily	1.18 (1.08–1.29), P < 0.001		0.75 (0.68–0.82), P < 0.001	
Three or four times a week	1.20 (1.09–1.32), P < 0.001		0.70 (0.63–0.78), P < 0.001	
Once or twice a week	1.19 (1.09–1.30), P < 0.001		0.83 (0.75–0.91), P < 0.001	
One to three times a month	1.44 (1.26–1.65), P < 0.001		0.80 (0.69–0.94), P = 0.005	
Special occasions only	1.37 (1.22–1.54), P < 0.001		0.73 (0.63–0.84), P < 0.001	
Never	1.29 (1.14–1.46), P < 0.001		0.74 (0.64–0.85), P < 0.001	
**Comorbidities**				
**Diabetes**		**0.053**		**0.009**
yes	1.27 (1.14–1.41), P < 0.001		0.67 (0.57–0.78), P < 0.001	
no	1.25 (1.19–1.31), P < 0.001		0.76 (0.73–0.80), P < 0.001	
**Hypertension**		**<0.001**		**<0.001**
yes	1.25 (1.17–1.32), P < 0.001		0.70 (0.65–0.75), P < 0.001	
no	1.26 (1.19–1.34), P < 0.001		0.80 (0.75–0.85), P < 0.001	
**CHD**		**<0.001**		**0.268**
yes	1.14 (1.04–1.24), P = 0.004		0.82 (0.74–0.91), P < 0.001	
no	1.29 (1.23–1.35), P < 0.001		0.74 (0.71–0.78), P < 0.001	
**CKD**		**0.327**		**0.803**
yes	1.20 (1.03–1.41), P = 0.023		0.80 (0.66–0.96), P = 0.020	
no	1.26 (1.20–1.31), P < 0.001		0.75 (0.72–0.79), P < 0.001	

Forest plots show HRs (95% CIs) for “Lower” vs “Higher” absolute HGS and ALM/height² (EWGSOP2) for associations with incident HF, based on segmented Cox regression with sex-specific inflection points (absolute HGS: 41 kg [males], 20 kg [females]; ALM/height²: 8.18 kg/m² [males], 6.45 kg/m² [females]). The “Higher” group served as the reference. Red vertical lines indicate the reference (HR = 1) at the inflection point. *P* for interaction was obtained from models including subgroup × sarcopenia indicator terms

Abbreviations: HR, hazard ratio; CI, confidence interval; HGS, handgrip strength; ALM, appendicular lean mass; BMI, body mass index; EWGSOP2, European Working Group on Sarcopenia in Older People 2.

Figure S6. Differential metabolite across sarcopenia phenotypes.

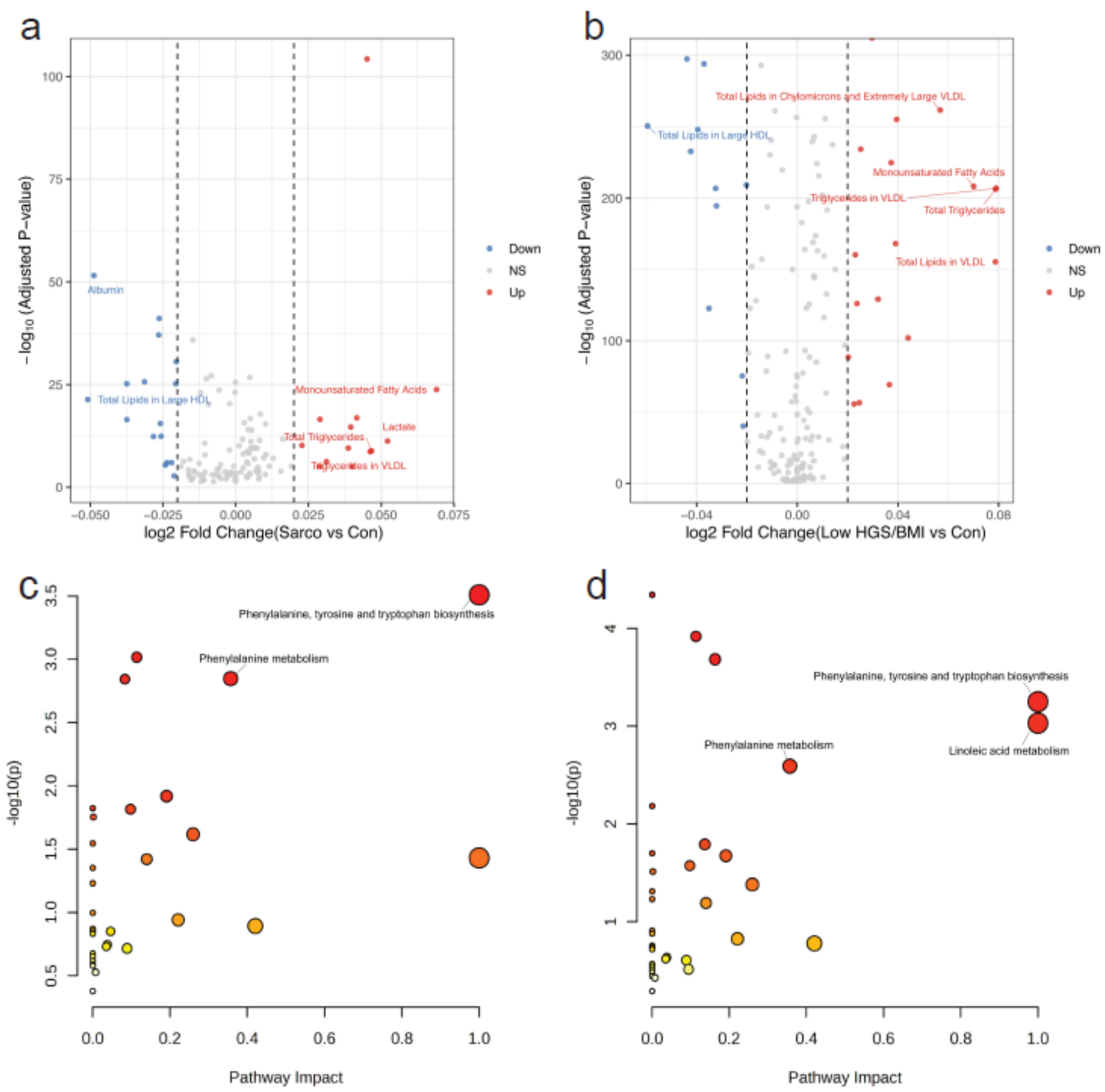

(a–b) Volcano plots of differential metabolites (|$\log_2$FC| > 0.02 and FDR < 0.05) between the (a) confirmed sarcopenia *vs.* reference and (b) low HGS/BMI only *vs.* reference. (c–d) Pathway enrichment of differential metabolites (FDR < 0.01) in (c) confirmed sarcopenia *vs.* reference and (d) Low HGS/BMI only *vs.* reference.

Abbreviations: Abbreviations: BMI, body mass index; HDL, high-density lipoprotein; LDL, low-density lipoprotein; NS, not significant; VLDL, very-low-density lipoprotein.

Figure S7. (a–b) Pearson correlation heatmaps of metabolites that were differentially expressed (FDR < 0.01) and significantly associated with incident HF in Cox models (Bonferroni-adjusted $P < 0.05$) in the (a) Confirmed sarcopenia and (b) Low HGS/BMI groups.
(c–d) Pearson correlation heatmap of metabolites that were differentially expressed ($|\log_2 FC| > 0.02$ and FDR < 0.05) and significantly associated with incident HF in Cox models (Bonferroni-adjusted $P < 0.05$) in the **(c)** *Confirmed Sarcopenia* vs. *Reference* comparison and **(d)** *Low HGS/BMI only* vs. *Reference* comparisons. Abbreviations: BMI, body mass index; FC, fold change; FDR, false discovery rate; HDL, high-density lipoprotein; HF, heart failure; HGS, handgrip strength; LDL, low-density lipoprotein; VLDL, very-low-density lipoprotein.

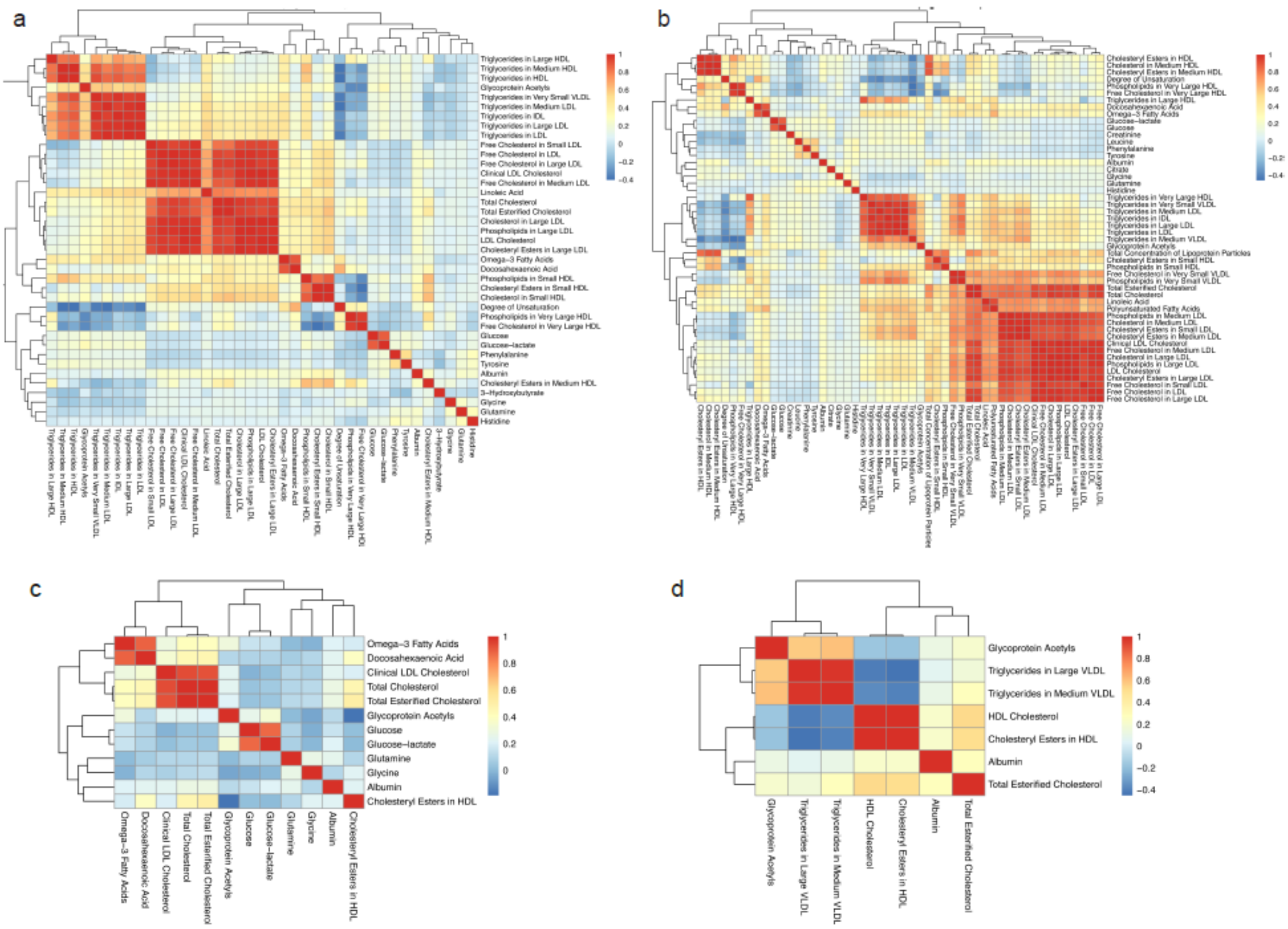

Figure S8. Calibration of 15-year heart failure risk prediction models.

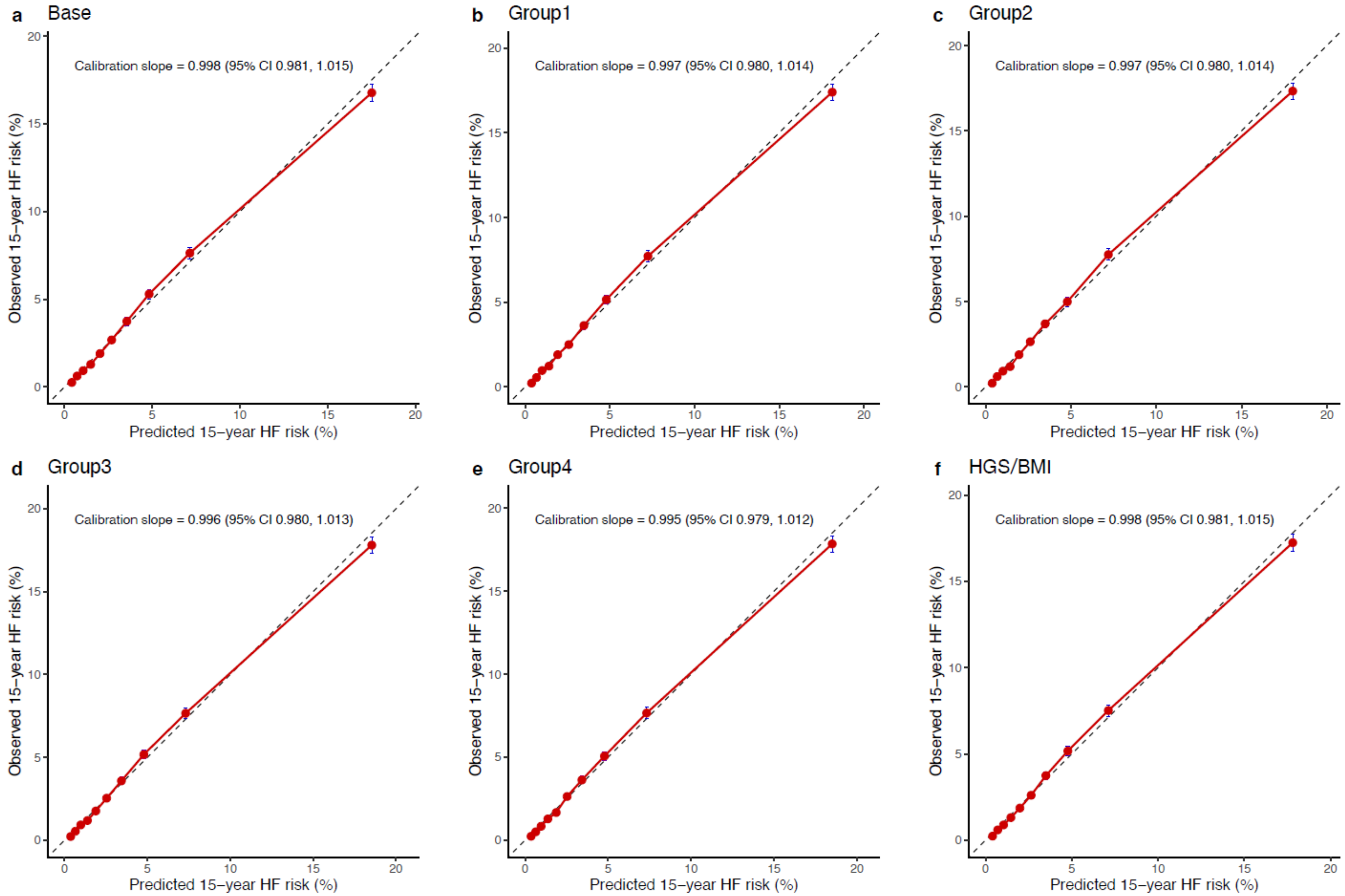

Calibration plots show observed versus predicted 15-year HF risk across deciles of predicted risk for the base clinical model (a), models additionally incorporating each LASSO-selected metabolite panel (b-e), and the model additionally incorporating HGS/BMI (low vs normal) (f). The dashed line indicates perfect calibration.

Abbreviations: BMI, body mass index; CI, confidence interval; HF, heart failure; HGS, handgrip strength; LASSO, least absolute shrinkage and selection operator.
